# Sodium alginate-based eco-friendly nanocomposites for textile wastewater management

**DOI:** 10.1039/d6ra06066h

**Published:** 2026-10-08

**Authors:** Amina Sajjad, Saher Goher, Hassam Mehmood, Sameera Sh. Mohammed Ameen, Aniqa Baig, Zahra Sajjad, Ali M. L. Usman, Khalid M. Omer

**Affiliations:** a Department of Chemistry, College of Science, Govt. College University Faisalabad Pakistan; b Department of Applied Chemistry, College of Science, Govt. College University Faisalabad Pakistan; c Department of Applied Chemistry, College of Science, Govt. College University Faisalabad Pakistan; d Department of Chemistry, College of Science, University of Zakho 42002 Zakho Kurdistan region Iraq Sameera.m.ameen@uoz.edu.krd; e Department of Chemistry, College of Science, Khwaja Fareed University of Engineering and Information Technology Rahim Yar Khan Pakistan; f Department of Zoology, College of Science, Government College University Faisalabad Pakistan; g Centre of Excellence in Solid State Physics, University of the Punjab Lahore 54000 Pakistan; h Department of Chemistry, College of Science, University of Sulaimani Qliasan St. 46002 Sulaymaniyah Kurdistan region Iraq khalid.omer@univsul.edu.iq

## Abstract

Increasing global textile production continues to intensify concerns about the discharge of untreated dye-laden wastewater into the environment. Textile effluents are typically dark in color and characterized by high pH, elevated biochemical oxygen demand, chemical oxygen demand, total dissolved solids, and total suspended solids. Moreover, textile dyes exhibit high levels of toxicity, carcinogenicity, genotoxicity, and mutagenicity toward living cells. In this regard, for environmental sustainability, there is a pressing need to improve the quality of effluents before they are discharged into water bodies. Therefore, various treatment techniques are being explored for textile wastewater, and many studies now use nanomaterials because their unique properties and easy customization make them highly effective for this purpose. Sodium alginate (SA) is an abundantly available, eco-friendly, and cost-effective biopolymer that can be effectively utilized as an adsorbent for wastewater treatment. Moreover, modifying SA or incorporating it into composites with other materials can significantly enhance its functional properties, broadening its potential for advanced environmental applications. In this review, we highlight the development and application of SA-based nanocomposites for effective textile wastewater treatment. Lastly, the review provides insights into the current limitations of SA-based materials and offers recommendations for future research directions to enhance their performance and applicability.

## Abstract 2

Increasing global textile production continues to intensify concerns about the discharge of untreated dye-laden wastewater into the environment.

## Graphical abstract

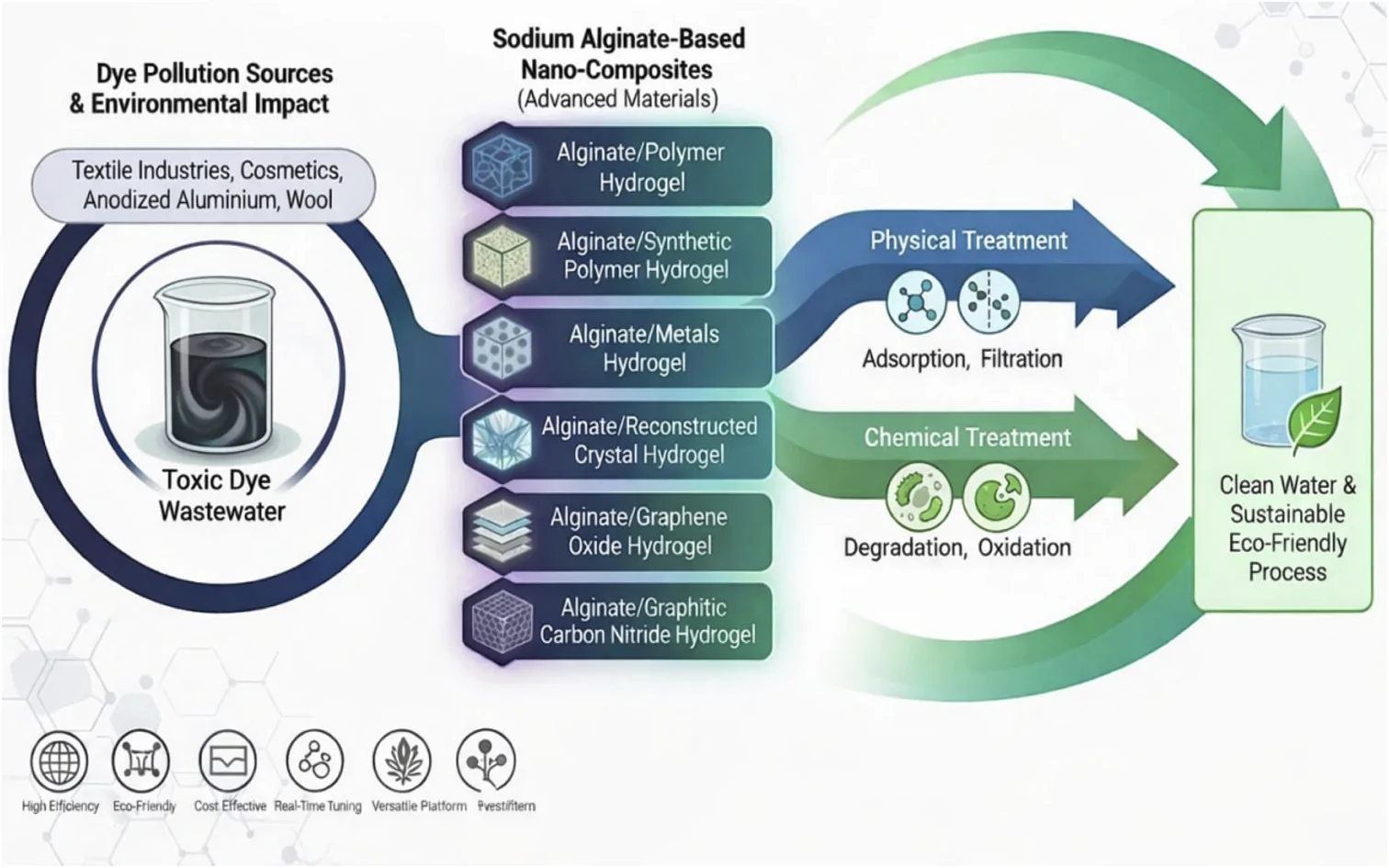

## 1. Introduction

Wastewater management has become a major global challenge, as rapid urbanization and industrial growth continue to elevate the volume of wastewater being produced, much of which is released into natural water bodies without adequate treatment.^1–6^ Therefore, there is an urgent need to develop advanced technologies that can effectively purify water and make it safe for use. Among these, nanotechnology-based approaches have emerged as a highly promising solution for treating contaminated water.^7–10^

Nanomaterials-based approaches represent rapidly evolving approaches in wastewater treatment, necessitating increased research focus and comprehensive understanding.^11^ Diverse nanomaterials are applied for the remediation of wastewater, with selection criteria dependent on the specific contaminants targeted and the desired treatment efficiency.^12^ Graphene, metallic and metal oxides nanoparticles, carbon-based nanomaterials, and metal organic frameworks are a number of the potential nanostructure for wastewater treatment.^13^ Nanosized ferric oxides can be used as cost-effective adsorbents to remove harmful metal ion from wastewater. Among these goethite, ferric oxides, hematite, magnetite and maghemite are extensively explored in this regard. Oxide based nano-particles including zinc oxide, titanium oxide, and magnesium oxide find widespread application in the field of wastewater treatment.^4^

Sodium alginate (SA), an environmentally friendly biopolymer sourced from brown seaweed, is highly effective in wastewater treatment due to its natural properties like biocompatibility and biodegradability.^14^ It is widely utilized as a scaffold for immobilizing materials such as nanoparticles, enzymes, or microbes, which significantly enhances the removal of challenging pollutants, including heavy metals and dyes.^15^ Its key feature is its ability to form a gel, forming high-surface-area materials (like beads or films) that efficiently capture contaminants. Furthermore, the material's performance can be easily optimized through chemical modification or by combining it with other nanomaterials, boosting its stability, selectivity, and overall adsorption capacity, making it a versatile and sustainable choice for modern water purification technologies.^16^

In this review, we are covering the utilization and efficacy of SA, a natural biopolymer derived from brown seaweed, as a cutting-edge material in wastewater treatment. Our primary focus will be on its key characteristics, biocompatibility, biodegradability, and remarkable adsorption properties, and how these enable its use as an immobilization matrix for enhancing the removal of pollutants like heavy metals and dyes. We will also detail the role of its gel-forming ability in creating high-surface-area capture materials (beads/films) and explore the various chemical modifications and nanocomposite combinations used to improve its stability, selectivity, and overall performance as a versatile, eco-friendly solution.

### 1.1 Water scarcity and toxicity of textile dyes

Water scarcity has become a major global challenge, as nearly three billion people worldwide lack access to clean drinking water. According to UNESCO's 2021 World Water Development Report, global freshwater use increased sixfold over the past century and has continued to grow at an annual rate of approximately 1% since 1980.^17^ According to the United Nations, by 2025, 2.7 billion people will not have access to clean drinking water^18^ By 2050, one-quarter of the world's population is expected to experience water stress due to water scarcity.^19^ According to the data from US Environmental Protection Agency the total storage of water resources is ≈1.386 × 10^9^ km^3^ although 70% surface is covered with water, only 3% freshwater is available for drinking purpose and only 0.06% is available pollution free. In 80 countries of the world, which contains 40% populations of the world about 1.5 billion people are facing drinking water shortage.^20^

Water pollution has been a major cause of deaths and spreading diseases all over the world. According to a report of Lancet Commission pollution was the root cause of 9 million premature deaths in 2015 which makes it the dominant risk factor for diseases and premature deaths. According to an updated data, pollution cause one in every six death globally.^21^ More than 80% of sanitary waste generated by human activities poured into water reservoirs without any treatment which is the cause of more than 50 different kinds of diseases. Poor water quality cause 80% of infections and 50% of child mortality.^17^ With the rapid growth of industrial activities, the discharge of effluents from sectors such as textiles, paper and pulp, pharmaceuticals, tanneries, paints, and bleaching operations has become increasingly alarming. Among all the above-mentioned industries, textile industries generate more than half (54%) of the total dye effluents followed by dyeing industries (21%), paper and pulp industries (10%), tannery and paint industries (8%) and dye manufacturing industries (7%). Dyes are used globally used having an annual production of 1 000 000 tons all over the world. Annually dyeing industries discharge approximately 7.5 metric tons of dyes.^22^

During the process of dyeing around 10–15% portion of dyes remains unbound and is directly discharged into water resources these sources of pollutants are shown in Fig. 1. Consequently 2–20% of used dyes are directly discharged into aquatic systems.^23^ These dyes are non-biodegradable they last longer in aquatic systems.

**Fig. 1:**
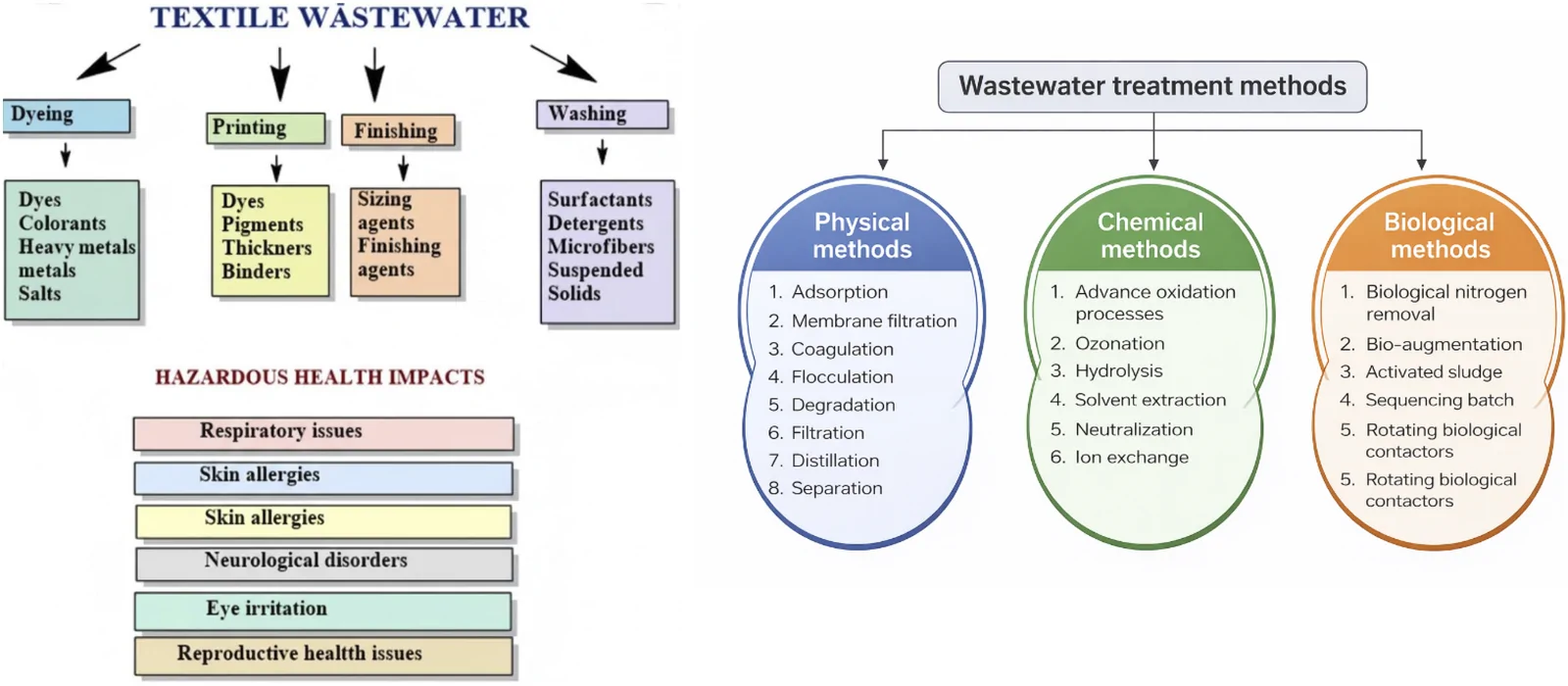
Schematic diagram of textile wastewater sources and types of pollutants.

Textile industry has a crucial part in advancement of economy of a nation. Due to rapid industrial development, the demand for synthetic dyes has grown significantly, with the textile sector alone accounting for approximately 50% of their consumption. The world's first synthetic dye, mauvine, was discovered by W. H. Perkin in 1856. Since then, the production and use of synthetic dyes have expanded extensively for a wide range of commercial applications. Dyes possess complex structures with aromatic rings linked to various functional groups containing π–electrons, enabling them to absorb light in the 380–700 nm range.^24^

### 1.2 Techniques for dye removal from wastewater

For centuries, drinking water was purified using medicinal plants, pebbles, sand, alum, or by simple filtration through muslin cloth.^25^ As water quality and composition continued to change, the need for more advanced treatment methods became increasingly apparent. Disinfectants such as chlorine and potassium permanganate were subsequently introduced to sanitize public water supplies, wells, recreational areas, and swimming pools. With the onset of industrialization, freshwater resources began to deplete rapidly, while the discharge of untreated industrial effluents progressively contaminated major water bodies, resulting in widespread disruption of aquatic ecosystems. To address these challenges, modern wastewater treatment relies on a range of advanced techniques, including adsorption, advanced oxidation processes (AOPs), membrane filtration (such as reverse osmosis and nanofiltration), biological treatment methods, and electrochemical processes, all of which aim to enhance contaminant removal and improve water quality all techniques for dye removal from wastewater shown in Fig. 2.^26^ Adsorption transfers contaminants from the liquid phase onto an adsorbent surface, effectively removing dyes, heavy metals, and organic pollutants. AOPs, in contrast, generate highly reactive species like hydroxyl radicals to degrade persistent organic compounds into harmless byproducts. Membrane filtration methods, such as reverse osmosis and nanofiltration, remove dissolved salts, organic compounds, heavy metals, and pathogens by selectively separating contaminants through semi-permeable membranes. Biological treatment methods use microorganisms to break down organic pollutants and nutrients, thereby reducing the overall pollutant load in wastewater. Together, these approaches provide efficient and complementary solutions, ensuring both pollutant removal and environmental protection.^27^

**Fig. 2:**
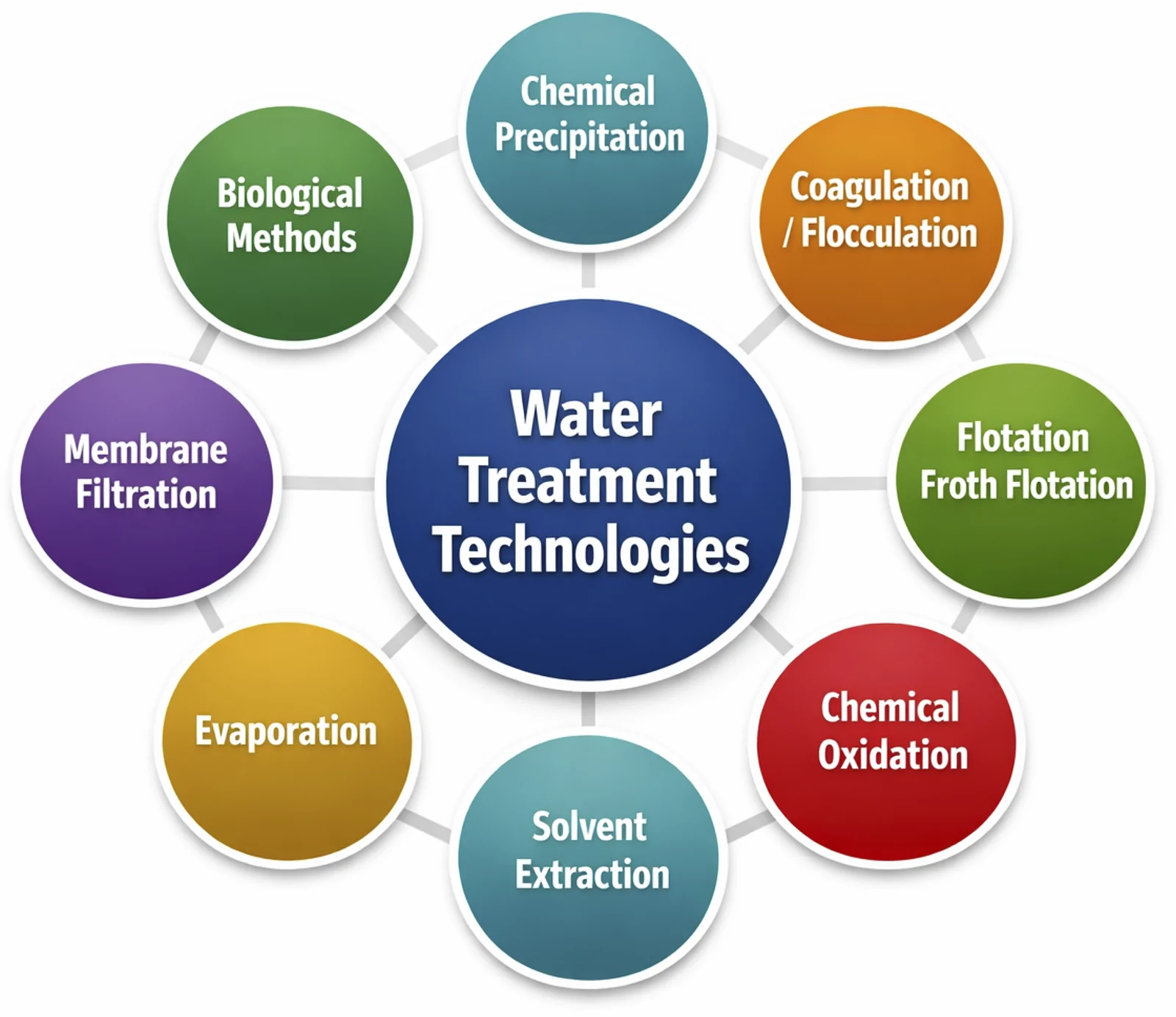
Techniques for dye removal from wastewater.

Unique characteristics of each treatment method may prove beneficial in certain aspects but also limited in others.^28^ The treatment methods which are costly, take more time to process, give less output or generate toxic byproducts are less likely to be considered are disacussed in Table 1.

**Table 1:** Comparative analysis of different wastewater treatment methods.^29,30^

No	Process	Merits	Limitations
1	Chemical precipitation	Technologically simple	Consumption of chemicals (lime, oxidants, H_2_S, *etc.*)
		Capable of handling high pollutant loads	Physicochemical monitoring of the effluent such pH levels
		Effective in significantly reducing the chemical oxygen demand	
2	Coagulation/flocculation	Simple process with low capital costs	Requires addition of non-reusable chemicals
		Efficient reduction in the chemical oxygen demand and biochemical oxygen demand	Leads to increased sludge generation
		Efficient removal of insoluble pollutants	
3	Flotation froth flotation	Efficiently removes small particles	Chemical consumption
		Capable of eliminating small particles	High cost
4	Chemical oxidation	High throughput	Chemical consumption
		No sludge generation	Ozone may form know intermediates
			Limited reduction in COD
5	Biological methods	Cost effective	Less effective in non-degradable and toxic pollutants
		Efficiently removes biodegradable organic matter	Process is slow due to kinetic limitations
		Effectively removes color and reduces suspended solids and biochemical oxygen demand	Low biogradability of dyes
6	Membrane filtration	Diverse applications	Investment costs are often too high for small and medium industries
		Multiple module configurations	
7	Evaporation	Versatile technique	High costs for large volumes of wastewater
8	Solvent extraction	An effective alternative to traditional lime precipitation for recovery of phosphoric acid	Requires significant investment in equipment
			Not cost effective when contaminants concentrations are less than 0.5 g L^−1^

Among all the methods available, adsorption is commonly regarded as economical and reliable method because it is technically simple and can be used for various contaminants.^29,31–34^ The efficiency of adsorption method can be reached up to 99.9% in removal of impurities. The United States Environmental Protection Agency (USEPA) has stated the adsorption as most effective and superior process for effluent treatment.^28^ Different types of adsorbent materials such as zeolites activated carbons, natural and synthetic polymers and clays have been suggested for the treatment of textile effluents.^35^

Ideally an adsorbent material should contain enough binding sites for facilitating effective adsorption. This high surface area can be obtained by using nano sized materials which can increase the area available for adsorption of pollutants.^36–38^ Nano scale materials are those materials which have one dimension in size range 1–100 nm. Materials of this range have high surface to volume ratio which results into more effective reactivity and adsorption towards various types of pollutants. Various physical and chemical properties of nano scale materials play a crucial part in selection of material. Many inorganic and organic nano scale materials have been employed for the treatment of waste.^39^ Organic pollutants are removed through adsorption, which occurs *via* van der Waals forces and various hydrophobic or electrostatic interactions with the adsorbent material.^40^

One of the interesting materials recently explored for dye removal is SA, a natural polysaccharide derived from brown seaweed, which is widely used in water treatment due to its excellent adsorption and flocculation properties.^41^ It can effectively remove heavy metals, dyes, and suspended solids from wastewater by forming gel-like networks that trap contaminants.^42^ Its functional groups, such as carboxyl and hydroxyl groups, interact with pollutants through ion exchange, chelation, and hydrogen bonding. Additionally, SA is biodegradable, non-toxic, and environmentally friendly, making it a sustainable option for both industrial and municipal water treatment applications.

### 1.3 Novelty and scope of review

Several recent reviews have discussed sodium alginate-based composites, hydrogels, and modified alginate materials for water and wastewater treatment. However, most of these studies have considered alginate-based materials from a broader water-treatment perspective, with less emphasis on their specific application to the complex wastewater generated by the textile industry. The present review therefore focuses specifically on sodium alginate-based nanocomposites for textile wastewater treatment, with particular attention to the removal of toxic textile dyes. It brings together recent research on the incorporation of different materials, including polymers, clays, metals, and metal oxides, into sodium alginate to improve its adsorption and treatment performance. In addition to summarizing the reported removal efficiencies, the review discusses the role of these modifications in improving the functional properties of alginate and examines the major mechanisms involved in dye removal. The advantages and limitations of these materials, together with issues related to stability, reusability, practical application, and future development, are also considered. In this way, the review provides a focused perspective on how sodium alginate can be modified into more effective nanocomposite materials for addressing the specific challenges associated with textile wastewater and identifies areas that require further research before these materials can be translated into practical treatment applications.

## 2. Alginate structure and physical properties

Alginate is primarily a linear polysaccharide made up of β-d-mannuronic acid and α-l guluronic acid as shown in Fig. 3 that are ordered in random and linked by α,β-(1,4)-glycosidic linkage.^43^ Alginate with diverse characteristics can be produced by arranging two monosaccharides in separate sequences.^44^ Alginate has certain characteristics, one of being its capacity to create hydrogels while divalent cations are present.^45^

**Fig. 3:**
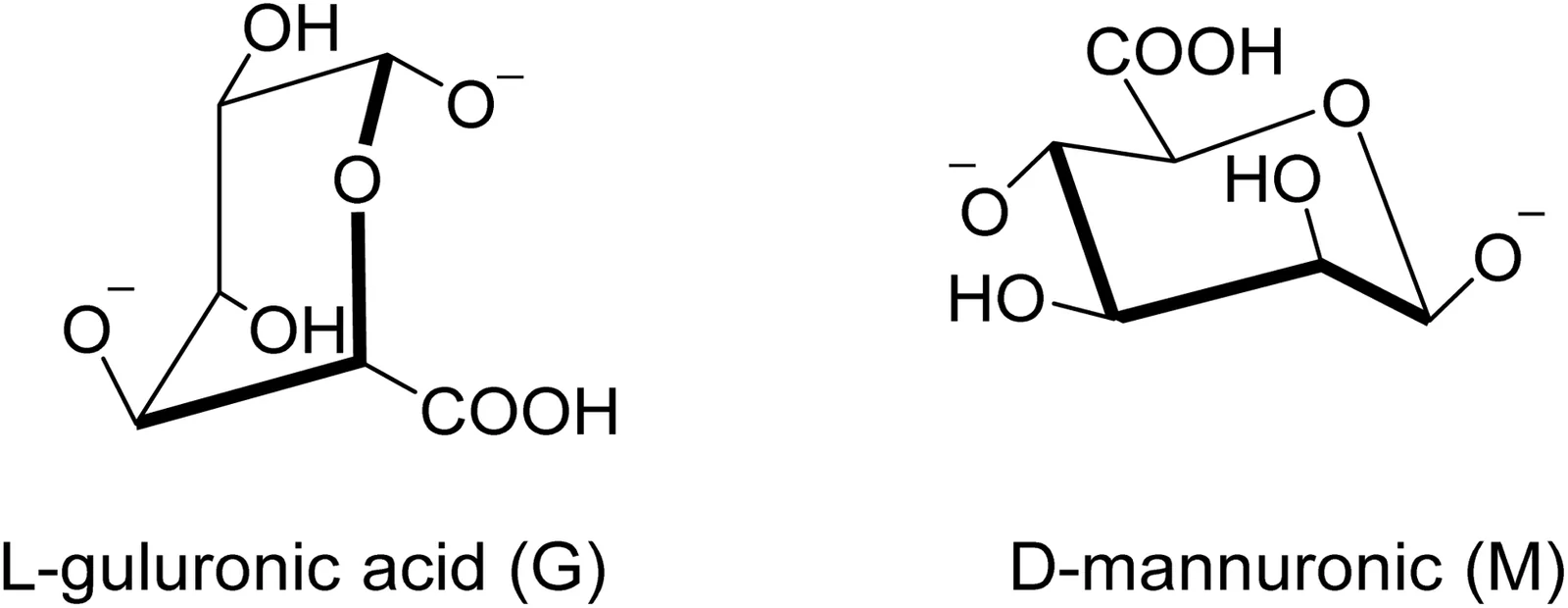
Alginate chemical structure of monomers: d-mannuronic acid and l-guluronic acid. Reproduced from ref. 46 with permission from Taylor & Francis, copyright 2017.

Polysaccharides, also referred to as hydrocolloids or natural gums, are a broad group of carbohydrate polymers composed of glycosidic linkages and derived from natural sources, as summarized in Table 2. They are generally classified into three main types: animal, plant, and microbial polysaccharides. These materials have been widely investigated for applications such as drug delivery systems, emulsifying and stabilizing agents, pharmaceuticals, and textile products.^47,48^ They have numerous functionalities in their organic structure, hydrogels based on gums adsorb pollutants from wastewater, such as dyes, heavy metal ions, and oils.^49,50^ The essential assistance of using material that extract from polysaccharides as adsorbents show ecofriendly behavior with environment and maximum gum polysaccharides are anionic exclusion of chitosan, which revenue that take away effectively cationic pollutants alike cationic dyes (*e.g.*, malachite green, methylene blue, rhodamine B)^51^ and heavy metal cations like Cu^2+^, Hg^2+^, Cd^2+^ & Pb^2+^.^52,53^ Though, improved polysaccharides similarly show potential for get rid of anionic pollutants, explicitly anionic dyes (*e.g.*, methyl orange and Congo red).^54–56^ SA, mechanically strong, or resistant to heat known for its biosafety, biocompatibility, and renewability^57^ due to SA's exceptional performance in terms of antibacterial activity, non-immunogenicity, gel formation capacity, and processing characteristics and due to its wide range of sources, mild preparation conditions, and low production costs.^58^

**Table 2:** Scientific literature survey for sources of SA

Sr No:	Source of SA	Analysis performed and results	Yield	Ref.
1	Tunisian seaweed	According to circular dichroism estimation of the mannuronic (M) and guluronic acids (G) value (M/G = 0.59), 37% of the composition of *C. barbata* SA was mannuronic acid and 63% was guluronic acid	9.90%	86
	*Cystoseira barbata*			
2	*Laminaris digitate*	M/G ratio 1.12	38.33%	87
	Brown seaweed	Viscosity 2.542 dl g^−1^		
		Average molecular mass: 1.14 × 105		
3	*Sargassum echinocarphum*	Average water content: 20.37–23.30%	Lowest yield in 5 h 2.78%	88
		Average ash content: 22.28–34.87%		
		Viscosity range: 18, 0–19.8 Cp		
4	*Dictyopteris membranaceae* & *Padina pavonica*	SA extracted from *D. membranaceae* contained 65% of uronic acid while this extracted from *P. pavonica* contained only 9% of uronic acid	18.93%	89
5	The three main brown seaweed varieties are *Sargassum aquifolium*, *Dictyota ciliolata*, and *Turbinaria triquetra*	Moisture content: 6.4–13.1%	(22.2 ± 0.56% DW)	90
		Ash content: 12.3–20% DW		
		Protein content: lipid concentration ranged from 0.3 to 3.5% DW, with a range of 0.57 to 1.47% DW		
6	*Cystoseira schiffneri*	Uronic acids: (47.4–66.4%) ash content: (24.3–39.4%)	32%	91
		Size: 4.49 to 1230 kDa		
		Thermogravimetric analysis		
		Stable until 400 °C		
7	Brown seaweed	Moisture content: 16.82%	31.70%	92
	*Sargassum wightii*	Ash content: 5.20%		
8	Brown algae *Sargassum aquifolium* & *Sargassum cinereum*	SEM/EDS analysis showed layer structure and fibre fibrils	Not reported	93
		Elements present: C, O, Na, Cl		
		A three-step process for heat deterioration. Beginning at 41.7 °C, which is related with dehydration as well the first stage was followed by 206.4 and 241 °C, which are connected with breakdown		

Recent studies have focused on modifying alginate materials to enhance selective adsorption, including the removal of dyes, heavy metal ions, aromatic compounds, phosphates, and the recovery of precious metals. A search of the ISI database identified approximately 800 articles on sodium alginate modification, with 107 published in 2022 alone—representing an 8.2-fold increase compared to 2001. The modified SA is used for dye removal, heavy metal ion removal, aromatic compound removal, phosphate removal, and precious metal recovery.^59^ Compared with other carbohydrate polymers, SA uniquely undergoes crosslinking with divalent cations (such as Ca^2+^, Ba^2+^, and Mg^2+^) without the need for a crosslinking agent, resulting in a three-dimensional network formed through interactions between the cations and α-l-guluronic acid blocks.^60,61^ A wide range of alginate-based and alginate-derived composite materials—such as graphene oxide–calcium alginate systems, calcium alginate/activated carbon and nickel alginate composites, graphene oxide aerogels, and sodium alginate–acrylic acid/graphite hydrogels—have been extensively developed and investigated for the adsorption and removal of organic dyes and other pollutants from wastewater.^51,62^

SA exhibits strong compatibility with a wide range of anionic substances, possesses excellent film-forming properties, and is completely biodegradable.^63^ However, films made from pure SA are highly water-soluble and generally show limited mechanical strength along with weak antimicrobial effectiveness. These drawbacks restrict their direct application in packaging materials, indicating that modification or incorporation of additional components is required to enhance their performance and functionality.^64,65^ A fundamental property of alginates is their interaction with multivalent metal cations, which induces crosslinking. As a result, SA exhibits decreased hydrophilicity, enhanced water barrier properties, and reduced water solubility.^66–68^ Cross-linking also leads to a marked enhancement of mechanical properties, while the incorporation of suitable plasticizers increases film flexibility. Together, these modifications broaden the range of potential applications for the films.^69–71^

### 2.1 Significance of SA

SA is an inexpensive and easy-to-obtain contagious control material with upright biocompatibility.^72,73^ To improve the hydrophilicity and mechanical strength of the material, frequently mixed with inanimate materials or additional polymer organic substances and prepare a polymer carrier through a network structure, which has not only high mass transfer efficiency and mechanical strength but also long service life.^74^ The demand for environmentally benign and efficient coagulant aids is steadily growing; however, the application of sodium alginate in combination with aluminum sulfate for water treatment has not yet been reported. It is anticipated that integrating SA with alum could improve coagulation performance while enabling a reduction in the required aluminum sulfate dosage.^75^ SA can be cross-linked to form a stable gel by using divalent metal ions as cross-linking agents. Sodium alginate (SA) is an anionic polysaccharide characterized by linearity and consisting of β-d-mannuronic acid (M) and α-l-guluronic acid (G) units organized in homopolymeric MM and GG blocks and heteropolymeric MG blocks.^76^ Alginate gelation process takes place due to the ionic interactions of carboxylate groups of alginate with multivalent cations. The most recognized model of Ca^2+^-induced alginate gelation is the “egg-box model” suggested by grant *et al.* Alginate gelation depends greatly on both type of cross-linking cation and molecular composition of polymer. While SA interacts with various divalent cations such as Ca^2+^, Ba^2+^, Sr^2+^, and Zn^2+^, the binding strength, selectivity, and gel formation differ from cation to cation. Thus, classical egg-box model must be mainly related to the Ca^2+^-induced G-block cross-linking rather than being applicable to all divalent ions.^77,78^ Mg^2+^ demonstrates considerably lower interaction with G-blocks compared to Ca^2+^ owing to high hydration ability and other characteristics of ion.

Alongside the crosslinking cation, the M/G ratio is another significant structural parameter affecting the physicochemical and mechanical behavior of alginate gels.^4^ The alginate having a higher G-block content usually has a stronger affinity towards calcium ions, which results in stiff, strong, and rigid gels due to the presence of favorable binding sites for ionic junction formation. On the other hand, M-rich alginate usually produces softer, more flexible and elastic gels since M-blocks bind less effectively with Ca^2+^ ions than G-blocks. Hence, an increase in the G content usually leads to an increase in the strength and rigidity of gels while an increase in M content increases their flexibility and elasticity.^77,78^

The M/G ratio and distribution affect not only the properties of alginate hydrogel but also its porosity, swelling, diffusion and mass transfer properties.^5^ G-rich alginates tend to produce crosslinked and mechanically robust networks while M-rich alginate usually has greater flexibility and swelling. It is especially important in case of alginate utilization as an adsorbent or immobilization matrix due to the influence of crosslinking on the availability of functional groups and penetration of contaminants into the polymer structure.^79^

SA can be crosslinked into the stable gel using divalent metal cations as cross-linking agents. The widespread oxygen-containing groups (*e.g.*, –OH and –COONa) in SA structure attain chemically modification. Helping after its hydrophilicity, biocompatible, salt tolerance, nontoxicity, abundance, and cost-effective properties^80^ the SA based-composites have countless claim by way of the adsorbent aimed at the removal of impurities. While laboratory studies on synthetic dye solutions dominate the sodium alginate (SA) literature, a smaller but growing body of work has tested SA-based materials directly on textile industry effluent—a more demanding matrix due to its high salinity, elevated COD/BOD, and complex mixture of contaminants. These studies offer a more realistic picture of SA's practical treatment potential. One of the earlier efforts in this direction immobilized dead *Candida tropicalis* cells within a sodium alginate matrix and applied the biosorbent in a packed-bed column fed with genuine textile effluent (pH 7.30, COD 1693 mg L^−1^, BOD 856 mg L^−1^, TDS 5765 mg L^−1^, TSS 3768 mg L^−1^). Best performance was obtained at pH 5 with a 15 cm bed height and a flow rate of 0.5 mL min^−1^, and the breakthrough behavior was well captured by the modified Thomas model—indicating that column-scale prediction is feasible for this system.^81^

Overall, the performance of SA-based adsorbents depends on how their structural features influence the accessibility and stability of the adsorption sites. Crosslink density and M/G composition affect swelling, porosity, mechanical strength, and the availability of –COO^−^ and –OH groups, while surface charge and nanofillers can further influence dye interactions and mass transfer. Higher crosslinking may improve stability but can limit swelling and dye diffusion, whereas a more swollen network may provide better access to adsorption sites but lower mechanical stability. Therefore, adsorption performance results from a balance between site accessibility, structural stability, and mass transfer. Reported adsorption capacities should also be compared carefully because pH, dye concentration, adsorbent dosage, temperature, and contact time can strongly affect the results. This provides a more realistic structure–property–performance view of how the egg-box structure and M/G composition influence SA-based adsorbents.

Chemical modification of alginate has also been explored to boost simultaneous removal of multiple contaminants. Beads formed from polyethyleneimine-grafted calcium alginate were tested against Remazol Brilliant Blue R dye and phosphate, reaching over 90% dye removal in a first regeneration cycle under model conditions; when the same beads were applied to actual textile wastewater, they still achieved roughly 80% color removal alongside 90% phosphate removal, with the process best fit by a Freundlich isotherm and second-order kinetics.^82^ This dual dye/nutrient removal capability is notable given that phosphate is rarely addressed in SA-adsorbent studies focused solely on color.

Beyond pollutant removal, some SA-based systems have targeted resource recovery. A double-network hydrogel combining sodium alginate with a superabsorbent polymer was designed not just to strip dye from effluent but to allow the captured dye to be recovered and reused in subsequent dyeing operations. In fixed-column trials, each gram of material captured 439 mg of Reactive Blue 4, following Langmuir-type equilibrium and pseudo-first-order kinetics—and critically, the recovered dye retained enough integrity to be reused industrially, pointing toward a circular-economy application of SA composites rather than simple pollutant capture.^83^

Magnetic functionalization has addressed a separate practical concern: adsorbent recovery and reuse. A cobalt ferrite–alginate nanocomposite was applied to a binary dye mixture representative of mixed-dye textile discharge (Reactive Red 195 and Reactive Yellow 145), where the magnetic core allowed the spent adsorbent to be recovered with an external magnet and reused across five treatment cycles without redesigning the separation step.^84^

Most recently, a sodium alginate/bentonite composite was tested on effluent sourced directly from an industrial textile park (Rooma Textile Park, India), rather than laboratory-simulated wastewater. Removal efficiency scaled with adsorbent dose, plateauing near 86%, and—unusually for this literature—the study tracked a full panel of wastewater quality indicators (COD, BOD, TDS, TSS, sulfate, chloride) rather than color removal alone, giving a more complete picture of treatment impact on effluent quality.^85^

Among those considered to be best for applications demanding high oxygen barrier characteristics is alginate. SA is one of the many biomolecules that has tremendous promise for the creation structural adsorbent because of its low cost, high bioavailability, variety of functional groups, and outstanding ease of processing. *Via* hydrogen bonding, electrostatic interactions, and/or coordination, the many hydrophilic oxygen-encompassing units (–OH/–COO–) found across the ALG molecule function as binding locations that have the ability to immobilize water contaminants.^94^ For the absorption of dyes from polluted sources, functioning aerogels derived from ALG are being widely employed as environmentally benign adsorbents.^95^

SA films have favorable properties like homogeneity, biological compatibility, openness, and fat- and oil-impermeability. But because SA is hydrophilic, its layer is less moisture-resistant than that of synthetic plastics, which means that its water-barrier qualities are not as good. Furthermore, SA film is not suitable for high carrying loads due to its mechanical characteristics. Diverse methods have been investigated to get over the restrictions of SA films. These consist of the use of cross-linking methods, adding nanofillers, mixing with other polymers, and treating with acids. Using cellulose nanocrystal (CNC), an organic and biodegradable nanofiller, is one of these tactics that shows promise.^96^

When discussing intelligent the adsorption it is important to recognize the exceptional qualities of natural biomass sources like chitosan (CS) and SA. Involved the coupling of montmorillonite and cellulose nanofiber with CS, which produced a gel-like absorbent with exceptional efficacy to remove copper dioxide, lead, and cadmium from effluent. Jiang and coauthors created a unique adsorbent by grafting polyacrylamide and graphene oxide upon SA. The main goal of this process was to make it easier for copper dioxide and Pb^2+^ ions to become adsorbent. When compared to adsorption agents that utilize just CS or SA separately, the previously described composites offer more advantageous properties like improved mechanical strength, higher stability of the solution, and easily adjustable shape. But in these composition systems, the main functional groups that facilitate the absorption of metals are the amino group in salicylic acid and the carboxyl group in CS.^97^

## 3. Sodium alginate (SA) and its modification

SA, a low-cost, bio-based polysaccharide, is widely used to build hydrogels and aerogels for dye-contaminated wastewater. Composites and crosslinking strategies greatly enhance capacity, stability, and reusability compared with pure SA. Sodium alginate (SA) can be structurally engineered using contemporary chemical and biochemical methods to generate novel alginate derivatives with well-defined monomer sequences and architectures, or by copolymerizing and grafting it with other polymers. Such modifications adjust key physicochemical properties—such as hydrophilicity, mechanical strength, and gelation behavior—allowing the material to be customized for targeted uses in food, biomedical, and environmental applications.^98^ One effective strategy is hydrophobic modification. In this approach, SA is rendered more hydrophobic by covalently attaching poly(butyl methacrylate) chains to the alginate backbone. The poly(butyl methacrylate) segment is first synthesized *via* free-radical polymerization of butyl methacrylate using 2-aminoethanethiol as a chain-transfer agent, which introduces terminal functional groups suitable for coupling to alginate. The resulting graft copolymer exhibits significantly increased hydrophobicity and altered gel behavior, enabling improved encapsulation and sustained release of hydrophobic drugs compared with unmodified alginate.^99^

SA-based hydrogels and aerogels are highly effective adsorbents for a wide range of cationic and anionic dyes, with optimized composites reaching capacities in the 100–4000 mg g^−1^ range and good reusability. Performance depends strongly on composite chemistry, porosity, and solution pH. Aerogels offer faster kinetics and multi-pollutant removal, while hydrogels are often easier to fabricate and regenerate. Further work focuses on long-term stability, real-wastewater validation, and scalable, continuous-flow applications.^100^

Hydrogels (beads/monoliths, often Ca^2+^-crosslinked): SA combined with lignin, coffee waste, banana-leaf activated carbon, organo-clay, or graphite, as well as PEI, PVA, κ-carrageenan, or inorganic fillers (TiO_2_, Fe_2_O_3_), yields porous, high-swelling networks with abundant –COO^−^/–OH and often –NH_2_ sites.^101^

A wider variety of hydrogels may be used for *in vitro* testicular tissue culture, although producing homogeneous hydrogels with fixed geometries is challenging due to the cross-linking processes of most conventional hydrogels. For instance, alginate was once used in ionic cross-linking to produce hydrogels. Photocrosslinked hydrogels, which are rapidly crosslinked into gels by photoinitiators utilizing UV light, are emerging in the biomedical field with the advent of material science. By adding methacrylic anhydride, nearly any type of hydrogel may be transformed into an equivalent photocrosslinked hydrogel.

It can display polyanionic behaviour in an aqueous solution with a certain level of adhesion and mobility.^102^ With these features of SA and Chitosan in consideration, conjugating CS and SA on liposomes *via* electrostatic interactions could increase liposome stability more successfully than utilizing a single biopolymer. While both polymers have been used separately to modify liposomes, their combined usage in liposomal modification has received less attention. Consequently, there is a pressing need to develop efficient structure modification techniques to raise liposomes' physicochemical stability.^103^

In the presence of 1-ethyl-3-[3-(dimethylamine) propyl], SA reacts chemically with *o*-and *m*-aminobenzoic acids-carbodiimide hydrochloride (EDC) resulted in the biopolymer's amide derivatives forming, which may form viscous hydrogel systems with thixotropic properties. After gentle stirring, the soft gel that had developed transformed into a flowing liquid, making it appropriate for potential use in formulations for personal care and health products as a sprayable gel material or delivery method for tiny active compounds as shown in Fig. 4.^104^

**Fig. 4:**
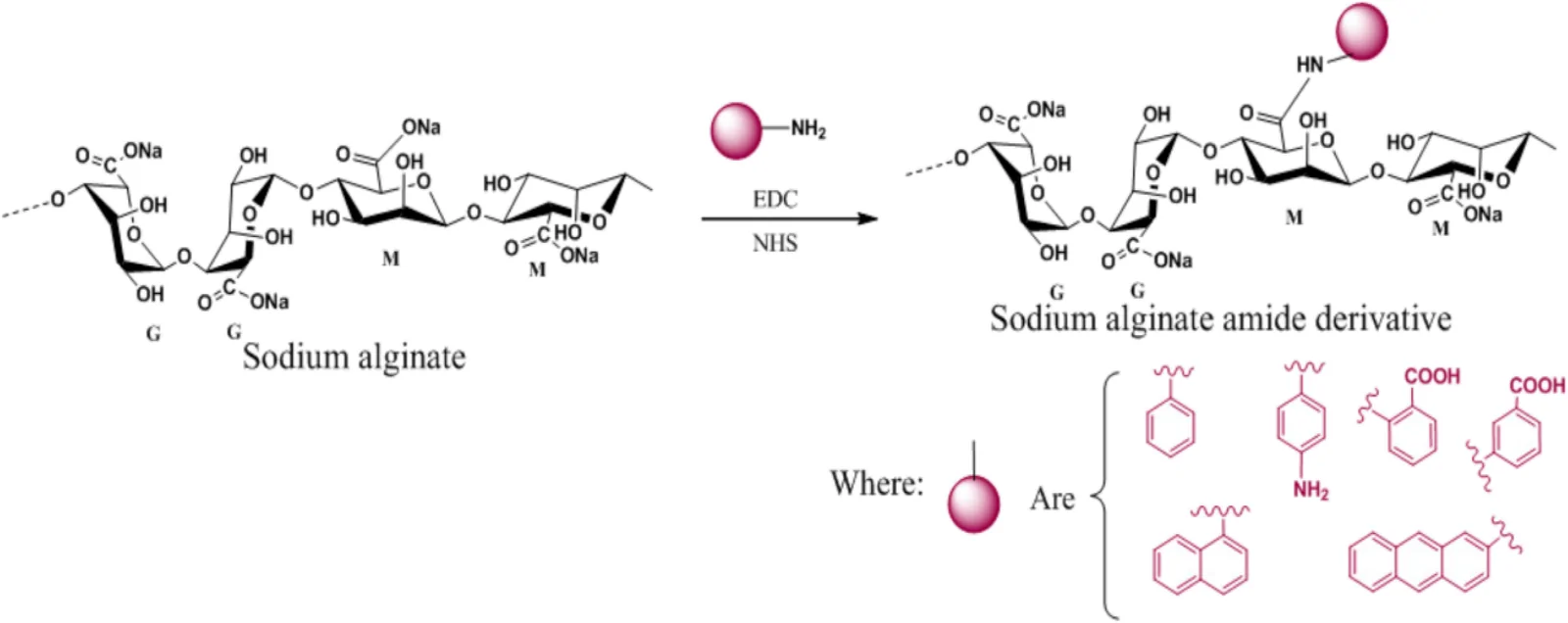
SA is chemically modified to produce a thixotropic hydrogel. Reproduced from ref. 104 under the terms of the Creative Commons CC BY licence, copyright 2021, the authors, published by MDPI.

SA-based aerogel contains abundant hydroxyl and carboxyl groups, providing polyanion active site for the adsorption of metals and cationic dyes. Freeze-dried SA composites with PVA/κ-carrageenan, chitosan, CNTs, cellulose, or chitosan/attapulgite form ultra-porous, low-density structures with >90% porosity and fast mass transfer, suitable for dyes and sometimes simultaneous metal removal.^105^

## 4. SA-based nanocomposites in wastewater treatment

Nanocomposites have recently become most prominent field in nanomaterial research.^106^ Because of unique properties of nanocomposites, *e.g.* high durability, rigidity and strength and resistance to corrosion, gases and heat, they offer many advantageous over conventional materials. Three major types of nanocomposites are reported in literature including metal matrix nanocomposites, ceramic matrix nanocomposites, polymer matrix nanocomposites (Table 3).^107^

**Table 3:** Literature survey for SA-based composites for dye removal

Sodium alginate-based composite and pollutant	Analysis/characterization	Removal efficiency/adsorption capacity	Kinetic/isotherm models	Ref.
**SA/polyacrylic acid/TiO**_**2**_ **hydrogel–methylene blue (MB)**	FTIR, SEM, TGA, XRD, TEM	**2257.36 mg g^−^** ^**1**^	PSO, Langmuir	113
**SA/flax-seed ash composite beads–MB**	FTIR, SEM, BET-BJH	**333.3 mg g^−^** ^**1**^ **; 90% removal**	PSO, Langmuir	114
**SA/graphite/polyacrylic acid hydrogel–malachite green (MG)**	FTIR, Raman, TGA, SEM, XPS, XRD	**628.93 mg g^−^** ^**1**^	PSO, Langmuir	115
**SA/polyaniline nanotube microbeads–methyl orange (MO)**	FTIR, SEM, XRD, TGA	**370.4 mg g^−^** ^**1**^	PSO, Langmuir	116
**Chitosan/SA composite foam–acid black 172 and MB**	SEM, FTIR, XRD, TGA	**817.0 and 1488.1 mg g^−^** ^**1**^, respectively	PSO, Langmuir	117
**SA/microcrystalline cellulose hydrogel–MO and MB**	FTIR, SEM, XRD	**331.25 and 253.31 mg g^−^** ^**1**^, respectively	PSO, Langmuir	118
**SA/gelatin/montmorillonite composite microbeads–crystal violet (CV)**	FTIR, SEM, XRD, TGA, BET	**1000 mg g^−^** ^**1**^ **; 92.1% removal**	PSO, Langmuir	119
**Cationic cellulose nanofibril/SA beads–Congo red (CR)**	SEM, FTIR, XRD, XPS	**518.4 mg g^−^** ^**1**^ **; >70% after 3 cycles**	—	120
**SA/polypyrrole nanotube composite beads–MB**	FTIR, SEM, XRD, TGA, BET	**666.7 mg g^−^** ^**1**^	PSO, Langmuir	121
**SA/flax-seed ash composite beads–MB**	FTIR, SEM, BET-BJH	**333.3 mg g^−^** ^**1**^ **; 90% removal**	PSO, Langmuir	114
**SA/graphite/polyacrylic acid hydrogel–malachite green (MG)**	FTIR, Raman, TGA, SEM, XPS, XRD	**628.93 mg g^−^** ^**1**^	PSO, Langmuir	115
**SA/polyaniline nanotube microbeads–methyl orange (MO)**	FTIR, SEM, XRD, TGA	**370.4 mg g^−^** ^**1**^	PSO, Langmuir	116
**SA/polyaniline nanocomposite–MB, rhodamine B, orange II, MO**	FTIR, SEM, TEM, XRD, TGA	**555.5, 434.78, 476.19 and 416.66 mg g^−^** ^**1**^, respectively	PSO, Langmuir	122

The efficiency of nanomaterials as purifiers has been confirmed for reducing total suspended solids, chemical oxygen demand^108^ biochemical oxygen demand,^109^ and harmful substances by processes which include advance oxidation processes,^110^ filtration,^111^ adsorption, and photocatalysis.^112^

Despite the potential of SA as an efficient adsorbent material, it has limitations in practical applications *e.g.* poor recyclability, limited sorption capacity and efficiency and poor recovery. Recently, SA based composites have been gaining increasing attention, which offer significantly improved performance over the use of single natural materials.^123^

Various nanofillers can be incorporated into biopolymer matrices, including nanoclays, nanoparticles, carbon nanotubes. Schematic diagram for dye removal address below in Fig. 5.^124^

**Fig. 5:**
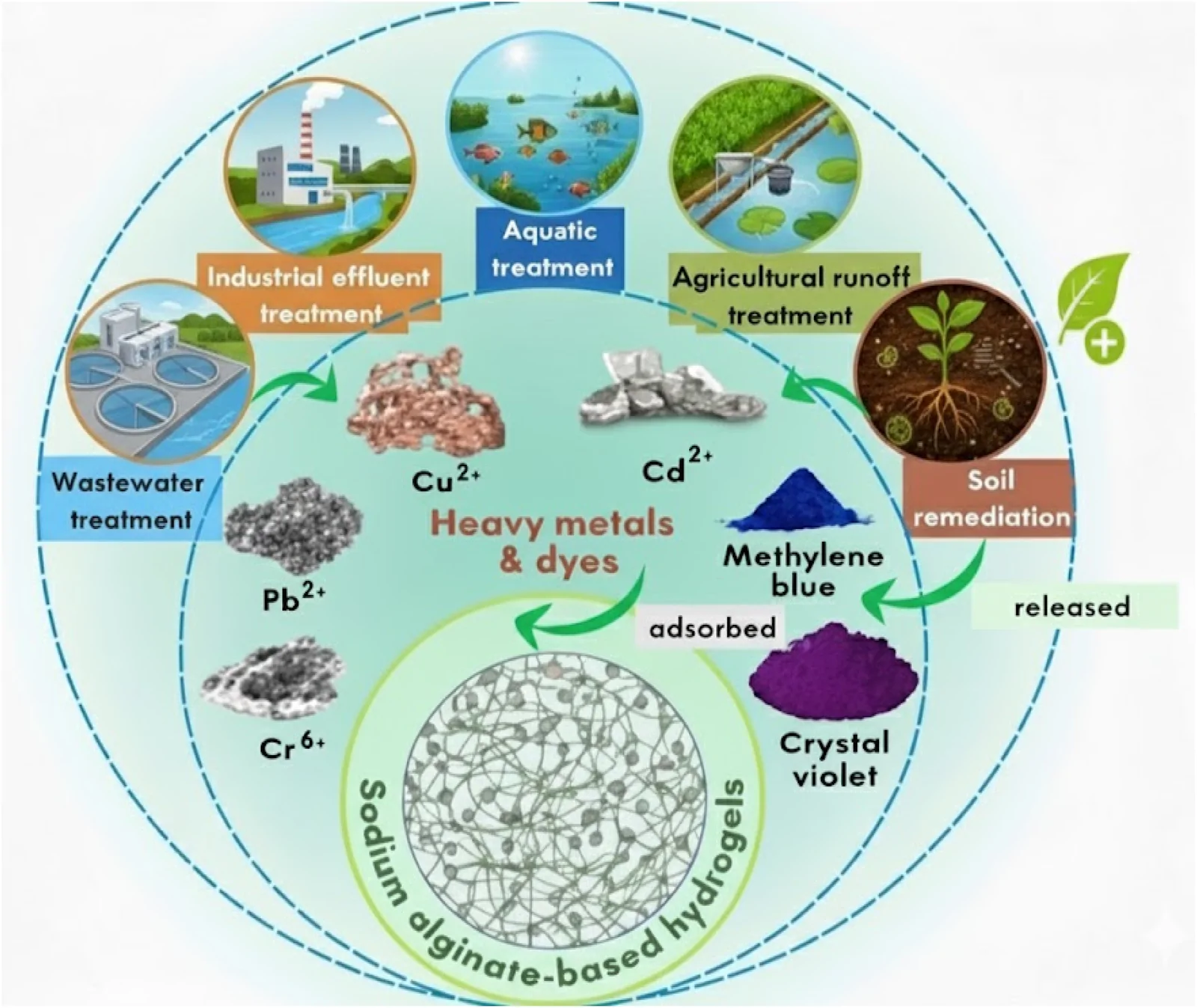
Schematic diagram for dye removal.

### 4.1 Sodium alginate/polymer nanocomposite

Polymer nanocomposites, particularly those involving SA, have gained significant attention for their effectiveness in dye removal from aqueous solutions. When combined with specific polymer types, SA exhibits synergistic effects that enhance its dye-removal capabilities. One common polymer used in conjunction with SA is polyvinylpyrrolidone (PVP). A study showed how to develop an innovative nanocomposite of SA, PVP, and TiO_2_ to remove cationic dyes from aqueous solutions.^125^ This composite showed promising cycling ability and reusability, making it a viable method of treating cationic dye-contaminated wastewater.

Another polymer commonly paired with SA is polyvinyl alcohol (PVA). Prepared composite nanofiber membranes of SA and polyvinyl alcohol for the adsorption of dyes such methyl blue, basic fuchsin, and methylene blue.^126^ The optimized nanofiber membranes exhibited excellent adsorption properties, highlighting the synergistic effects of combining SA with PVA.

Furthermore, the use of polyaniline in combination with SA has been studied for dye removal. Developed a SA and polyaniline nanocomposite with pH-responsive adsorption capabilities for the removal of dyes from water.^122^

This study further confirms that it enhanced adsorption capabilities achieved through the synergistic effects of polyaniline and SA.

In summary, the synergistic effects observed when SA is combined with polymers such as PVP, PVA, and polyaniline result in composite materials with improved dye removal efficiency. These polymer nanocomposites offer promising solutions for the treatment of wastewater polluted with various dyes, showcasing the potential of combining different polymers with SA for enhanced adsorption capabilities.

### 4.2 Electrospinning SA nanofibers

Electrospun nanofibers have gained significant attention for their uses in textile wastewater treatment due to their exceptional characteristics. Electrospinning enables the fabrication of composite membranes, which combine nanofiber layers with microfiber structures, improving the overall performance of the membrane.^127^

These nanofibers are advantageous as they have high porosity, small pore size, and good connectivity, making them highly suitable for filtration applications.^128^ In addition, electrospun nanofibers have high surface area to volume ratio, their controllable physical properties, and their capability to fabricate complex nanostructures are other key features for multiple uses such as wastewater treatment.^129^

In the textile wastewater treatment, these fibers have been utilized for the removal of toxic ions and organic pollutants using physisorption, chemisorption, and electrostatic attraction mechanism.^130^ The high specific surface area, and large porosity of electrospun nanofiber materials contribute to their effectiveness in wastewater treatment by providing large surface area for adsorption and filtration processes make them a versatile media in numerous wastewater treatment applications.^131^ Electrospun polymer micro/nanofiber membranes are an appealing alternative for a variety of wastewater treatment applications due to their high surface-to-volume ratio, excellent porosity, and linked pore architectures.^132^

The electrospun nanofibers exhibit many more associated characteristics than their physical properties. They are eco-friendly, for example, requiring no organic solvents and able to be used to produce submicron fibers from molten polymers. This makes them a good fit for a variety of biotechnological applications.^133^ Limitations still exist for the utilization of electrospun nanofibers in wastewater treatment, particularly with regard to pristine polymeric nanofibers. Research is ongoing, and in future, it may further enhance the efficiency of electrospun nanofibers in treating wastewater.^134^ Overall, electrospun nanofibers offer a versatile and effective solution for textile wastewater treatment because of their exceptional properties, including high porosity, controllable pore sizes, and high surface area-to-volume ratios. These characteristics make them valuable for the elimination of pollutants and toxins from wastewater, showcasing their potential in addressing environmental challenges in the textile industry.

### 4.3 Crosslinked sodium alginate nanocomposites

Crosslinked sodium alginate nanocomposites are essential in water treatment. They are fabricated by incorporating different crosslinking agents into SA matrices, resulting in improved properties that are beneficial for water treatment processes by eliminating pollutants, heavy metals, and other contaminants from water. For instance, the incorporation of multivalent cations, such as calcium ion or calcium sulfate, as crosslinkers is demonstrated to greatly enhance the alginate hydrogels increasing their efficiency to eliminate a wide range of contaminants such as ionic dyes.^135^

Moreover, the incorporation of nano-materials, such as graphene oxide or montmorillonite, into SA matrices, followed by crosslinking, generates advanced nano-composites with improved mechanical and chemical properties, making them well-suitable for water treatment.^136–138^ Crosslinking SA with various agents, not only enhances the mechanical properties of the nano-composites produced but also impacts their water absorption and swelling behavior, which are critical factors in water treatment.^139^

The ability to produce innovative materials is also demonstrated by the successful preparation of degradable and antibacterial SA nanocomposite films including functional components, such as Au–TiO_2_ nanoparticles, which can be used for advanced water treatment packaging.^138^ The controlled release characteristics of SA-based nanocomposites, achieved through crosslinking with various agents, also render them suitable for applications necessitating precise delivery of active substances in water treatment processes.^140^

In conclusion, crosslinked SA nanocomposites exhibit enhanced properties such as mechanical strength, controlled release capabilities, and improved stability, making them valuable materials for diverse water treatment applications. The incorporation of different crosslinking agents into SA matrices enable the customization of properties to meet specific requirements in water treatment processes, highlighting the multi-functionality and potential of these nanocomposites in addressing water treatment challenges.

### 4.4 Sodium alginate/clay nanocomposites

Clay based nano-composites have gained greater focus in past few years due their abundant availability, economic efficiency, efficient adsorption and high surface area.^141,142^ The lamellar structure of clay results in greater surface area which in turn increases its capacity for adsorption. Clays are naturally present aluminosilicate substances which contain trace amounts of organic compounds and metallic ions. Clay surfaces have negative charges that allow them to eliminate cationic dyes like methylene blue.

However through the modification of clays they are enabled to remove anionic dyes as well. Clays have been extensively used to remove metal ions and textile dyes.^143^ Incorporation of clay minerals in polymeric systems can enhance the surface properties of polymeric systems for modern applications and for pollutant removal from water.^144^ Even small amount (<5%) of incorporation of clay in polymer material can increase the properties quickly.^145^

Dye elimination from wastewaters using SA-containing clay nano composites have been reported by many researchers SA and clay minerals have been widely employed in the treatment of actual effluents as well as the removal of dye from aqueous solutions. By combining SA a natural polymer with clay, advantageous characteristics of both the materials are combined which in turn increases the functionality of system for the adsorption purpose. A hybrid material like clay has a porous structure and a lot of surface area, so it's easy for it to adsorb through noncovalent interactions like van der Waals forces, hydrogen bonds, electrostatic interactions, hydrophobic interactions and electrostatic interactions. Meanwhile, SA acts as a binder or stabilizer.^143^

Here we will review hybrid systems containing clay and SA which have been used for the elimination of textile dyes (2020–2023).

### 4.5 Sodium alginate metal/metal oxide nanocomposites

SA in addition with metal or metal oxide has been frequently used to remove hazardous pigments. SA in combination with metal oxide has synergistic effect of both photocatalysis and adsorption. Such type of materials are bifunctional materials in which one part acts as an adsorbent and the other one (metal oxide) acts as photocatalyst.^146^ Due to their diverse surface chemistry, adjustable band edges, distinct crystalline structure, unique texture, exceptional stability and precisely controlled morphologies metal oxide nano-materials exhibit remarkable efficacy in the elimination of organic pollutants from water. In past few years, interest is growing in metal enhanced photocatalysis due to ability of metal to indirectly or directly degrade pollutants.^147^ Metal oxides including TiO_2_, CeO_2_, ZnO, FeO, NiO, SnO_2_, CuO nano-particles have been used in treatment of coloured effluents.^148^

Here we will review nano-composites of SA and metal or metal oxide for the treatment of colored effluents as shown in Table 4 (2020–2024).

**Table 4:** Scientific literature for SA/metal or metal oxide nano-composites for textile water treatment

Sr No.	Adsorbent and adsorbate	Analysis performed	Efficiency	Models	Ref.
1	Acrylic acid/sodiumalginate/Cu^2+^ ions/ammonia	FT-IR, SEM, TEM	96%	PSO, Freundlich	149
	**Pollutant**: crystal violet, malachite green		Reusability: upto 8 cycles		
2	SA/cerium oxide–stannous oxide	FT-IR, FESEM	95.16%	Not reported	150
3	**Pollutant**: methyl orange silver/nickel oxide/calcium alginate	FT-IR, XRD, SEM	96.54%	PSO	151
	**Pollutant**: rhodamine B, methyl orange		Reusability: upto 5 cycles		
5	SA/graphene oxide/tannic acid/guar gum	TA, FTIR, SEM	203 mg g^−1^	Freunlich, Langmuir	152
	**Pollutant**: malachite green				
6	Alginate–silver nanoparticle/mica	FTIR, SEM-EDX, TEM, XRD, TGA/DTA	249 mg g^−1^	PSO	153
	**Pollutant**: methylene blue, brilliant orange		352 mg g^−1^		
			Reusability 4 cycles		
7	SA/ZnO NPs	FT-IR, XRD, TEM, UV-vis	34.63 mg g^−1^	Langmuir, Freundlich, Temkin, D–R	154
	**Pollutant:** Cr(vi)				
8	ZnO/SnO_2_/SA nanocomposite	FTIR, PXRD, SEM, and PL	99%	Not reported	155
	**Pollutant:** Congo red				

## 5. Environmental impact and sustainability

SA as a biopolymer extracted from brown seaweds is being appreciated globally due to its environmental friendly properties and biodegradability which makes it superior to synthetic polymers. Because it is an organic compound, it can naturally biodegrade with no formation of toxic byproducts hence offering a solution towards cutting on the long-term pollution and thus fulfilling circular economy.^156^ Biodegradability of SA can be explained by its chemical formula that contain d-mannuronic & l-guluronic of blocks which can be easily attacked by microbial action.^157^

This property is particularly of advantage in situations like wastewater treatment where SA composites can capture nanoparticles for improving adsorption for contaminants without affecting the environment once the materials are treated.^158^ When in composite form, SA can include a variety of nanoparticles within its structure and thereby improve mechanical and adsorption characteristics. For example, when SA is reinforced with magnesium oxide nanoparticles, the resulting property enhancement makes the material suitable for instance, in tissue engineering application.^159^ In the same way, SA, through its ionotropic gelation characteristic, is capable of creating stable nanoparticles that can encapsulated drugs or contaminants hence enhancing the effectiveness of the drug delivery systems and efficiency and effectiveness of pollutant gettering systems.^160^

The allowance of SA to form hydrogels and nanoparticles through interaction with cations add another advantage of the compound to many uses.

The evaluation of the effectiveness of the methods used in treating the textile wastewater shows different results between the traditional techniques and composites based on SA. Traditional systems, such as chemical coagulation, and activated sludge, are described as costly procedures that use a significant amount of energy and the release of secondary pollutants in the form of sludge that needs to be dumped.^161,162^ These methods commonly use toxic reagents, which not only raises the operating expense but also lead to several environmental concerns regarding secondary products.^163,164^ However, though chemical coagulation is effective in the removal of dye, it does not destroy the dye particles, thus the possibility of developing even more toxic by-products.^163^

While some of them are listed below, composites prepared from SA are relatively more sustainable. These composites rely on use of marine renewable resources, hence; no use of fossil fuel hence reducing the carbon footprint of the products. According to life cycle assessments, SA composites generate comparatively less carbon footprint than synthetic materials such as polyacrylamide which are used in conventional water treatment processes. By incorporating these bio based materials, the global sustainability agenda of resource recovery from waste is boosted.^165^

Moreover, the novel application of SA composites in wastewater treatment is backed by their comparatively lower energy demand in the fabrication process. In comparison with traditional methods that typically require a large amount of energy to be spent on chemical reactions or mechanical processes, the application of SA composites is more energy efficient in their production and application.^166^ For this reason, the described treatment method can be regarded not only environmentally friendly but also sound economically for industries that work to minimize their impact on nature.^167^

Besides, through the use of SA composites in wastewater treatment, it is possible to recover additional resources from the wastewater, including unexhausted dyes as well as inorganic salts which could be reused for subsequent dyeing processes.^166^ This resource recovery perspective is increasingly recognized as essential in sustainable wastewater management, as it transforms waste into valuable inputs rather than viewing it solely as a disposal challenge.^165^

## 6. Advantages and potential challenges

Some of the benefits associated with SA-based composites include which include; functional groups, heavy metals and dyes as well as organic pollutants may be adsorbed by SA based composites due to their tunable nature.^168^

Further, they are reusable as well as cheap and securable in different forms such as beads or hydrogels.^168^ However, opportunities also come with certain limitations: natural polymers have a lower mechanical strength compared to synthetic polymers and thus their use may be restricted in high pressure systems.^169^

Moreover, the increase of the scale of production of these kinds of composites for industrial uses is rather difficult owing to the differences in the quality of the starting components and the demand for conducting special technological processes.^170^

## 7. Applications

SA nanocomposites formed from SA have become the latest development for efficient elimination of pollutants in textile wastewater especially dyes and heavy metals. The application of functional nanoparticles; iron oxide, TiO_2_, carbon nanotubes and graphene oxide into SA matrices has been important in increasing the adsorbing and degrading properties of these gels.

### 7.1 Sodium alginate@carbon nano-particles

Shirani *et al.*^171^ investigated a composite material consisting of graphitic carbon nitride nanosheets (g-C_3_N_4_NS) and the biopolymer alginate for the removal of Congo red (CR) dye from aqueous solutions that shown in Fig. 6A. To overcome limitations associated with using calcium alginate or g-C_3_N_4_NS alone—particularly difficulties in recoverability—silica-coated magnetic nanoparticles (SMNPs) were incorporated into the composite. The alginate-based bioadsorbent was prepared using an *in situ* method, in which SA was crosslinked with calcium chloride in the presence of g-C_3_N_4_NS and SMNPs. This composite was then applied for CR dye adsorption, and the effects of key operational parameters—initial dye concentration, contact time, adsorbent dosage, and solution pH—were systematically evaluated. Isotherm analysis revealed that the linear Langmuir model provided the best fit to the experimental data, indicating monolayer adsorption. The maximum adsorption capacity for CR reached 68.03 mg g^−1^ at pH 5 within 120 minute.

**Fig. 6:**
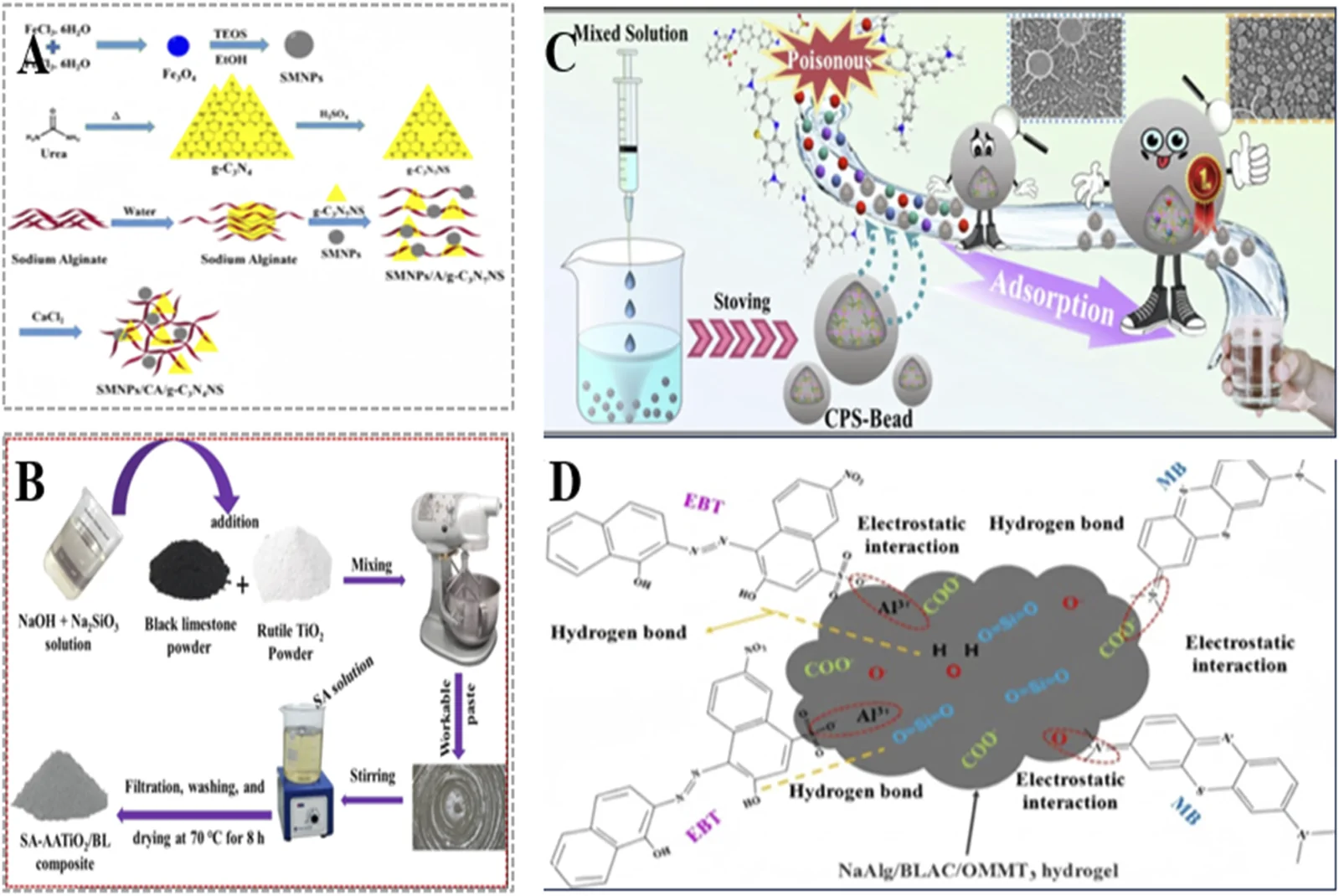
(A) Preparation of SMNPs/CA/g-C_3_N_4_NS. Reproduced from ref. 171 with permission from John Wiley and Sons, copyright 2025. (B) Synthesis steps of SA-AATiO_2_/BL material. Reproduced from ref. 172 with permission from Elsevier, copyright 2025. (C) Schematic illustration of CPS-Beads for removing MB, MG, CV and CR from dye-contaminated wastewater. Reproduced from ref. 123 with permission from Elsevier, copyright 2024. (D) The proposed adsorption mechanism for MB and EBT onto NaAlg/BLAC/OMMT_3_ hydrogel. Reproduced from ref. 101 under the terms of the Creative Commons CC BY licence, copyright 2024, the authors, published by Springer Nature.

Alshehri *et al.*^172^ developed a novel biosorbent (SA-AATiO_2_/BL) by modifying a mixture of naturally carbonized limestone (BL) and rutile TiO_2_ nanoparticles with SA for the adsorption of methylene blue (MB) that shown in Fig. 6B. Physicochemical characterization revealed that the resulting material possessed abundant surface functional groups and enhanced adsorption properties. Adsorption studies showed that the pseudo-first-order kinetic model (PFO) and the Langmuir isotherm provided the best fit for the kinetic and equilibrium data, respectively. The maximum MB adsorption capacity reached 263.40 mg g^−1^ at 25 °C and increased to 283.42 mg g^−1^ at 55 °C. Parameters derived from the statistical monolayer model offered insights into adsorption behavior, including the number of functional groups involved, active site density, interaction energies, and saturation capacities. The findings suggested a multimolecular adsorption mechanism with vertically oriented MB molecules at all tested temperatures. Thermodynamic analysis indicated that the adsorption process was endothermic and spontaneous, driven primarily by physical interactions such as hydrogen bonding and electrostatic forces, with interaction energies ranging from 16.80 to 17.53 kJ mol^−1^. Regeneration studies over six adsorption–desorption cycles demonstrated that SA-AATiO_2_/BL maintained good stability and reusability, highlighting its potential for real-world wastewater treatment applications.

Chen *et al.*^173^ incorporated carbon nanotube-wrapped polyacrylamide (CP) particles into SA to fabricate CPSA hydrogels featuring tortuous microchannels. This structural design allows the hydrogel to maintain an optimal balance between photothermal conversion and water transport by simultaneously enhancing its photothermal efficiency and extending the water migration pathway that shown in Fig. 6C. Under 1.0 sun irradiation, the CPSA hydrogel achieves a high evaporation rate of 1.97 kg m^−2^ h^−1^. In addition to its solar-driven water evaporation performance, the CPSA hydrogel exhibits strong photocatalytic activity for wastewater treatment, including effective degradation of methylene blue (MB). The tortuous internal architecture also endows the 6 mm-thick CPSA hydrogel with exceptional electromagnetic interference (EMI) shielding effectiveness, reaching 81.1 dB primarily *via* absorption mechanisms. Overall, this study offers valuable insights into designing multifunctional hydrogels that support water–energy balance management while providing high-performance electromagnetic wave shielding.

Arafa *et al.*^101^ reported the development of advanced sodium alginate-based hydrogels designed for water treatment applications by integrating activated carbon derived from banana leaves with organo-modified montmorillonite clay. The activated carbon was successfully synthesized from banana leaf biomass, while the montmorillonite was chemically modified using cetyltrimethylammonium bromide, a cationic surfactant, to enhance its compatibility with the polymer matrix. The composite hydrogels were subsequently prepared through ionic cross-linking with calcium chloride, resulting in a stable hydrogel structure, as shown in Fig. 6D. The resulting SA hydrogels exhibited strong dye-removal capabilities, achieving removal efficiencies of up to 80.3% for methylene blue (MB) and 84.9% for eriochrome black T (EBT) within 90 minutes at pH 7. Adsorption behavior aligned more closely with the Freundlich isotherm model. EBT removal followed pseudo-second-order kinetics, whereas MB removal was best described by a combination of pseudo-first-order and intraparticle diffusion models.

Cytotoxicity was assessed using MTT assays, which revealed a dose-dependent decline in cell viability. Among the tested materials, SA-based hydrogels showed the lowest cytotoxicity. Overall, the findings indicate that the developed hydrogels are safe and effective candidates for water treatment applications.

Makhado *et al.*^174^ prepared a multifunctional hydrogel nanocomposite by integrating sodium alginate with poly(acrylic acid) and oxidized multi-walled carbon nanotubes through an *in situ* free-radical polymerization approach. This composite material was designed for wastewater treatment and evaluated for its ability to adsorb methylene blue from aqueous media. Comprehensive characterization confirmed the successful formation and structure of the nanocomposite. The hydrogel showed exceptional swelling behavior, reaching 2265.4% at pH 8.0. Batch adsorption studies were conducted to examine the influence of critical factors, including solution pH, adsorbent amount, interaction time, and initial dye concentration. Adsorption kinetics were best described by a pseudo-second-order model, while equilibrium data conformed well to the Langmuir isotherm, indicating monolayer adsorption. At 25 °C, the material achieved a notably high adsorption capacity of 1596.0 mg g^−1^, demonstrating efficient dye removal as well as good reusability and regeneration stability. In the study by Li *et al.*^123^ a simple and efficient strategy was employed to synthesize SA gel bead adsorbents (CPS-Beads) featuring a porous architecture and high adsorption capability. The incorporation of polyamine-modified 3D carbon materials (GO-CNT) further enhanced the structural and functional properties of the gel beads. The optimized adsorbent was subsequently used to remove methylene blue (MB), malachite green (MG), crystal violet (CV), and Congo red (CR) from dye-contaminated wastewater. The CPS-Beads exhibited exceptionally high adsorption capacities, reaching 2191.39 mg g^−1^ for MB, 2201.69 mg g^−1^ for MG, 1669.11 mg g^−1^ for CV, and 2096.04 mg g^−1^ for CR at 318 K. Adsorption kinetics aligned well with the nonlinear pseudo-second-order model, while the Langmuir isotherm provided the best fit for equilibrium behavior. Thermodynamic analyses indicated that adsorption of all four dyes occurred spontaneously. Furthermore, the CPS-Beads demonstrated excellent recyclability, selectivity, and continuous adsorption performance in cyclic, selective-adsorption, and column experiments, confirming their potential for practical wastewater treatment applications.

Momina *et al.*^175^ synthesized a magnetic SA biocomposite embedded with carbon dots (CDs) for the removal of environmental pollutants, including dyes and metal ions. The CD-modified nanoparticles were efficiently integrated into the biopolymer matrix, enhancing both adsorption capacity and regeneration performance through a cost-effective and environmentally sustainable process. Comprehensive characterization confirmed that the CD-functionalized nanoparticles exhibited good dispersion, rich surface functional groups, and excellent adsorption behavior. Process parameters influencing adsorption were optimized using Response Surface Methodology (RSM), yielding a maximum adsorption capacity of 232.44 mg g^−1^ under optimal conditions. Additionally, an Artificial Neural Network (ANN) model with a 3-5-5-1 architecture was developed to predict the composite's adsorption capacity, demonstrating strong predictive accuracy. A statistical physical model was also applied to elucidate the adsorption mechanism and molecular orientation of dye species on the composite surface, estimating an adsorption capacity of 467.57 mg g^−1^. Column adsorption experiments confirmed the composite's strong adsorption efficiency and effective regeneration over repeated cycles. A detailed cost analysis further demonstrated the economic feasibility of the synthesized biocomposite for practical wastewater treatment applications (Fig. 7).

**Fig. 7:**
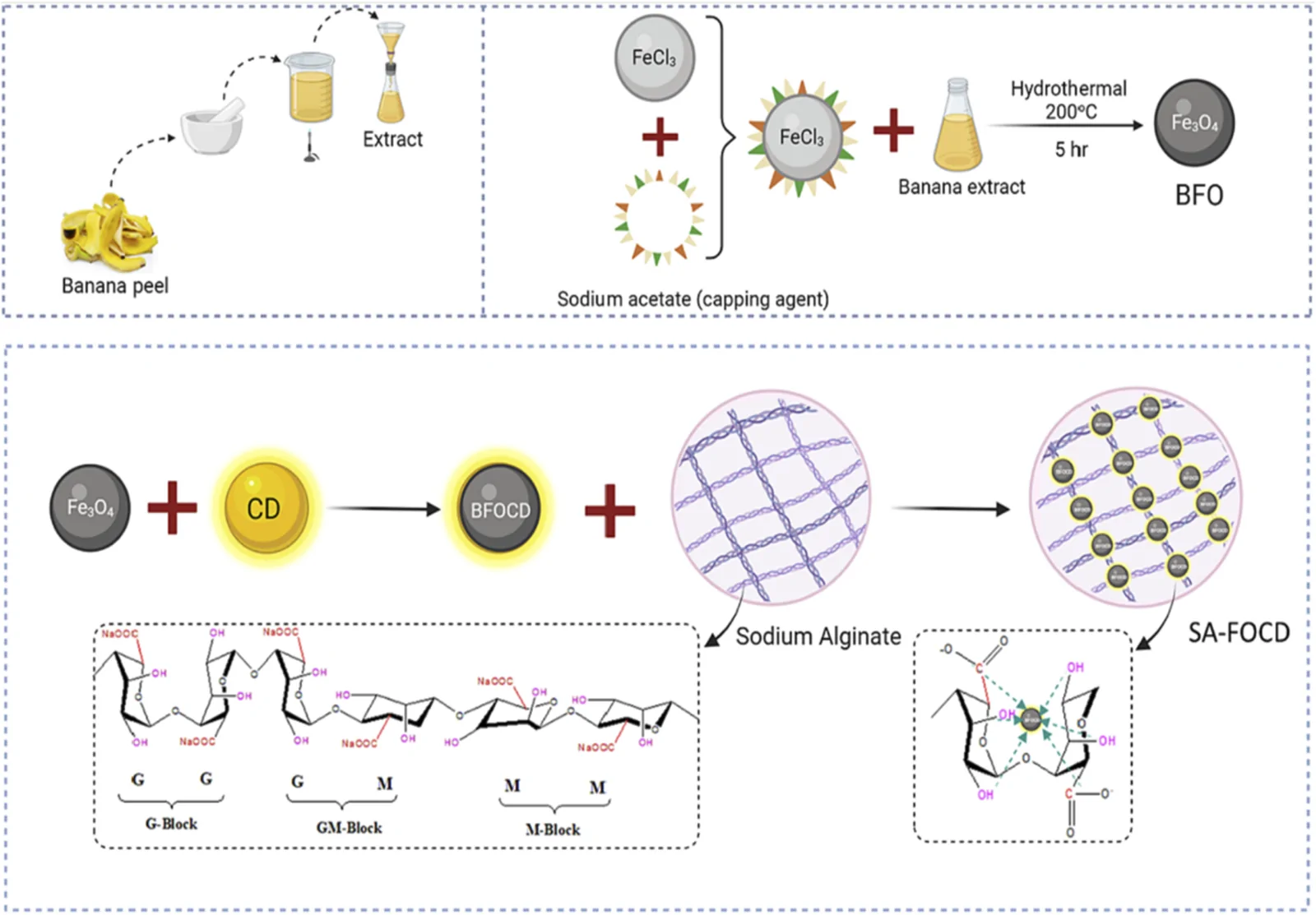
Synthesis procedure of iron oxide nanoparticles (BFO), and SA composite (SA-FOCD). Reproduced from ref. 175 with permission from Elsevier, copyright 2024.

An *et al.*^176^ fabricated CNT/SA aerogels with a three-dimensional porous network using sol–gel processing followed by freeze-drying that shown in Fig. 8. The resulting aerogels exhibited excellent adsorption performance toward cationic dyes, achieving a methylene blue (MB) adsorption capacity of 195.29 mg g^−1^—superior to many conventional adsorbents. In addition, the aerogels retained more than 80% removal efficiency after five adsorption–desorption cycles. These results underscore the strong potential of CNT/SA aerogels as efficient materials for dye wastewater purification.

**Fig. 8:**
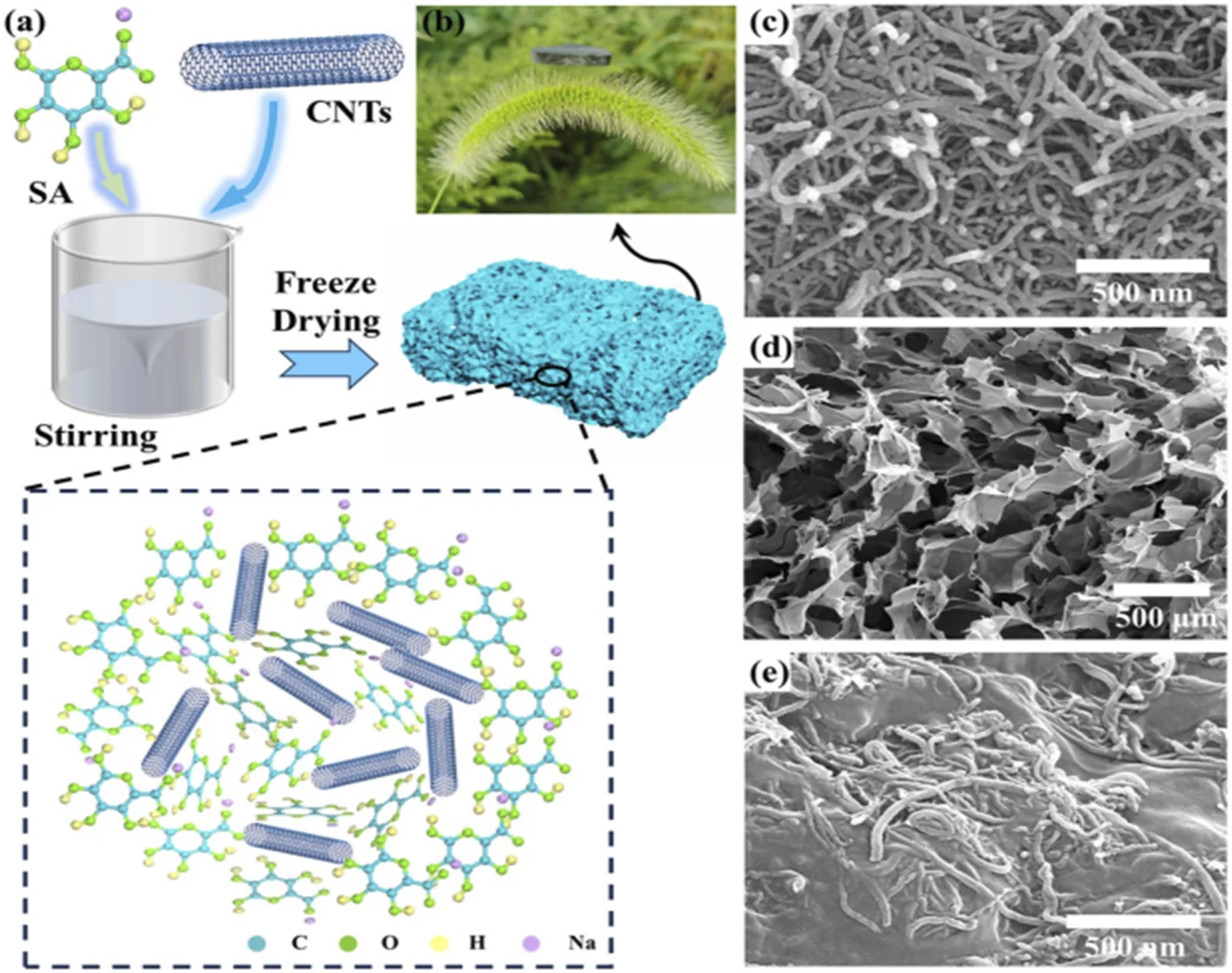
(a) Schematic illustration of the fabrication process for the porous CNTs/SA composite aerogel, (b) photographic image of the CNTs/SA aerogel positioned on a setaria, SEM images of (c) CNTs, CNTs/SA aerogel at (d) low magnification and (e) high magnification. Reproduced from ref. 176 with permission from John Wiley and Sons, copyright 2025.

Fatima Ilter *et al.*^177^ investigated the adsorptive performance of SA/carboxymethyl cellulose/activated carbon (SA/CMC/AC) ternary composite microbeads prepared with varying activated carbon (AC) loadings for the removal of methylene blue (MB) from aqueous solutions. Composite beads were synthesized by incorporating AC at 10%, 20%, and 30% (by weight relative to the polymer content), followed by crosslinking in a 2 wt% CaCl_2_ solution that shown in Fig. 9A.

**Fig. 9:**
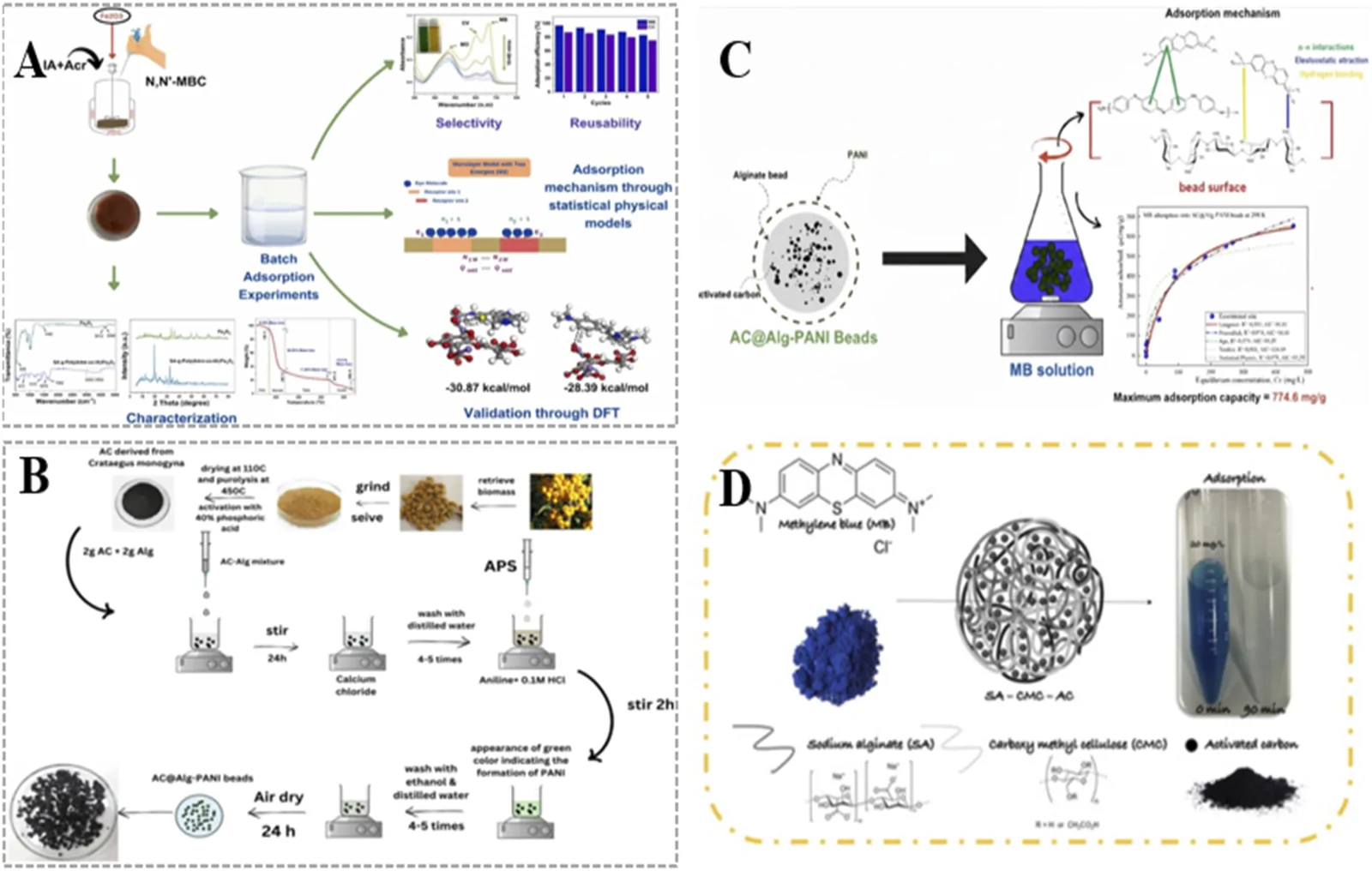
(A) Schematic illustration of SA/CMC/AC for the removel of MB. Reproduced from ref. 178 with permission from Elsevier, copyright 2024. (B) Preparation process of AC@Alg-PANI composite beads. (C) Schematic illustration of AC@Alg-PANI for the removel of MB. (B) & (C) Reproduced from ref. 179 with permission from Elsevier, copyright 2024. (D) Schematic illustration of NCH by incorporating different amounts of Fe_2_O_3_ nanoparticles, aiming to remove the MB and CV from water. Reproduced from ref. 177 with permission from Taylor & Francis, copyright 2024.

Preliminary adsorption studies identified the optimal conditions as pH 7 and 298 K, with the microbeads containing 30% AC exhibiting the highest performance. Equilibrium data were fitted to several isotherm models, with the Langmuir isotherm providing the best description of the adsorption behavior. Using this model, the maximum adsorption capacity was calculated as 308.6 mg g^−1^ at 298 K. Kinetic analysis revealed that MB adsorption followed a pseudo-second-order mechanism. FTIR-ATR spectra confirmed interactions between the dye molecules and the adsorbent, highlighting the role of hydroxyl and carboxyl groups as active adsorption sites. SEM images further verified the adsorption process by showing the presence of dye particles adhered to the bead surfaces.

Chaima *et al.*^179^ developed a novel composite bead adsorbent, AC@Alg-PANI, composed of activated carbon (AC) derived from *Crataegus monogyna*, sodium alginate (Alg), and polyaniline (PANI), and evaluated its performance for methylene blue (MB) removal. The composite's physicochemical properties were thoroughly characterized using pH_PZC_ analysis, FTIR, TGA/DTA, SEM, and BET surface area measurements. Additionally, the effects of key operational parameters—including initial pH, dye concentration, adsorbent dosage, ionic strength, and temperature—were systematically assessed. A full factorial experimental design was used to identify optimal adsorption conditions, which were determined to be pH 6, an adsorbent mass of 100 mg, and an MB concentration of 100 mg L^−1^. Isotherm analysis revealed that MB adsorption followed the Langmuir model, yielding a high maximum adsorption capacity of 774.6 mg g^−1^. Kinetic modeling indicated that the process conformed to the pseudo-first-order model, suggesting a predominantly physical adsorption mechanism. Thermodynamic parameters further confirmed that the adsorption was favorable, spontaneous, and endothermic. The AC@Alg-PANI beads also exhibited strong reusability, maintaining high removal efficiency after five regeneration cycles. Molecular dynamics simulations supported the experimental findings by showing that adsorption was driven by physically spontaneous interactions, including van der Waals forces, hydrogen bonding, intermolecular forces, and π–electron interactions that shown in Fig. 9B and C.

Azari *et al.*^180^ investigated the removal of dye (ADMI, American Dye Manufacturers' Institute) from raw textile wastewater using a magnetic multi-walled carbon nanotube-loaded alginate composite (CNT-Alg-Fe_3_O_4_). The effects of key operational parameters—pH, adsorbent dosage, and contact time—were modeled and optimized using a response surface methodology-based central composite face-centered design (RSM-CCF). The model demonstrated strong agreement with experimental results, as indicated by a very low *p*-value (7.97 × 10^−13^), a low RSD (1.121), and a coefficient of determination greater than 0.953. Under optimized conditions (pH 3, CNT-Alg-Fe_3_O_4_ dosage of 10 g L^−1^, and contact time of 85.55 minutes), the composite achieved a removal efficiency of 98.43% and an adsorption capacity of 138.97 ADMI g^−1^. Equilibrium data were best described by the Langmuir isotherm (*R*^2^ = 1; *χ*^2^ = 0.012), confirming monolayer adsorption, while the pseudo-second-order kinetic model (*R*^2^ = 0.993; *χ*^2^ = 0.015) indicated a chemisorption-dominated process. Thermodynamic parameters (Δ*H*° > 0, Δ*S*° > 0, Δ*G*° < 0) revealed that the dye removal was spontaneous and endothermic. The composite also demonstrated good reusability, with only an 11.94% reduction in removal efficiency after ten consecutive adsorption–desorption cycles. The adsorption mechanism was attributed to the abundance of negatively charged carboxyl and hydroxyl groups on the CNT-Alg-Fe_3_O_4_ surface, enabling interactions with dye molecules through electrostatic attraction and π–π dispersive interactions that shown in Fig. 9D.

Fathy *et al.*^181^ developed novel bio-polymeric bead adsorbents composed of polylactic acid/SA (PLA/SA) and polylactic acid/SA/carbon nanoparticles (PLA/SA/CNP), intended as environmentally friendly materials for dye removal. The beads were characterized using scanning electron microscopy and Fourier-transform infrared spectroscopy. Incorporating carbon nanoparticles enhanced the dye adsorption efficiency, with 5 wt% identified as the optimal CNP loading. The effects of adsorbent dosage, contact time, initial solution pH, and dye concentration on adsorption performance were thoroughly evaluated. Using 50 mg of PLA/SA/CNP beads, the highest methylene blue (MB) removal efficiency was achieved at pH 9 after 120 minutes. Adsorption data were fitted to both Langmuir and Freundlich isotherm models, with the Langmuir model providing the best description and indicating a maximum adsorption capacity of 304.88 mg g^−1^.

### 7.2 Sodium alginate@metal oxide nano-particles

Umar *et al.*^178^ developed a new SA-based hydrogel nanocomposite (NCH) by incorporating different amounts of Fe_2_O_3_ nanoparticles, aiming to remove the cationic dyes methylene blue (MB) and crystal violet (CV) from water. The hydrogel containing 5% Fe_2_O_3_ showed the highest removal efficiency for both dyes. Comprehensive characterization using spectroscopic, microscopic, and spectrometric methods confirmed the structure and adsorption properties of the nanocomposite. Batch adsorption experiments indicated that the best results were achieved at pH 9, using 20 mg of adsorbent per 100 mL, with contact times of 60 minutes for MB and 40 minutes for CV, and an initial dye concentration of 500 mg L^−1^. The adsorption mechanisms differed: MB was primarily adsorbed through physical interactions, while CV adsorption involved both physical and chemical processes, including electrostatic attraction and hydrogen bonding. Although the adsorption mechanisms varied, the equilibrium data for both dyes fit best to the Langmuir–Freundlich isotherm model, suggesting that monolayer adsorption predominated, even on the heterogeneous surface of the NCH. Further analysis using a dual-site M2 statistical physics model indicated that both dyes were adsorbed in a multi-molecular, vertical arrangement on the nanocomposite's binding sites, with site heterogeneity playing a role in dye uptake. Density functional theory calculations showed that MB had a stronger adsorption affinity than CV, mainly due to hydrogen bonding and electrostatic interactions. The NCH maintained about 80% removal efficiency after five reuse cycles, demonstrating its promise for treating industrial wastewater.

Umar *et al.*^182^ conducted research to create a robust and effective magnetic nanocomposite hydrogel (MNCH) designed for the targeted removal of methylene blue (MB) and crystal violet (CV) dyes. The hydrogels were synthesized with varying amounts of Fe_3_O_4_ (ranging from 0 to 9 wt%) using a graft co-polymerization technique involving SA, acrylamide, itaconic acid, ammonium persulfate, and *N*,*N*-methylene bisacrylamide. Among the samples, the hydrogel containing 5 wt% Fe_3_O_4_ (MNCH_5_) achieved the highest dye removal efficiency, exceeding 95%. The optimal conditions for dye adsorption were found at pH 10, with 40 minutes required for CV and 60 minutes for MB using a 30 mg sample. Characterization methods such as X-ray diffraction (XRD) and Brunauer–Emmett–Teller (BET) analysis revealed a crystallite size of 30.5 nm and a surface area of 59.80 m^2^ g^−1^, respectively. The adsorption process followed fractal pseudo-first-order and fractal Vermeulen diffusion models, indicating a heterogeneous adsorption mechanism, as shown by the fractal exponent (*h*) values between 0.38 and 0.44. The Langmuir–Freundlich isotherm provided the best fit for the adsorption data, highlighting a greater affinity of MNCH for MB (4216.69 mg g^−1^) compared to CV (3730.17 mg g^−1^). Thermodynamic analysis indicated that MB adsorption was exothermic (negative Δ*H*), while CV adsorption was endothermic. Density functional theory calculations further supported a stronger interaction between MNCH and MB (*E*_ads_ = −49.29 kcal mol^−1^) than with CV (*E*_ads_ = −41.30 kcal mol^−1^). Overall, these results demonstrate the high adsorption capacity of MNCH, making it a promising material for dye removal applications that shown in Fig. 10.

**Fig. 10:**
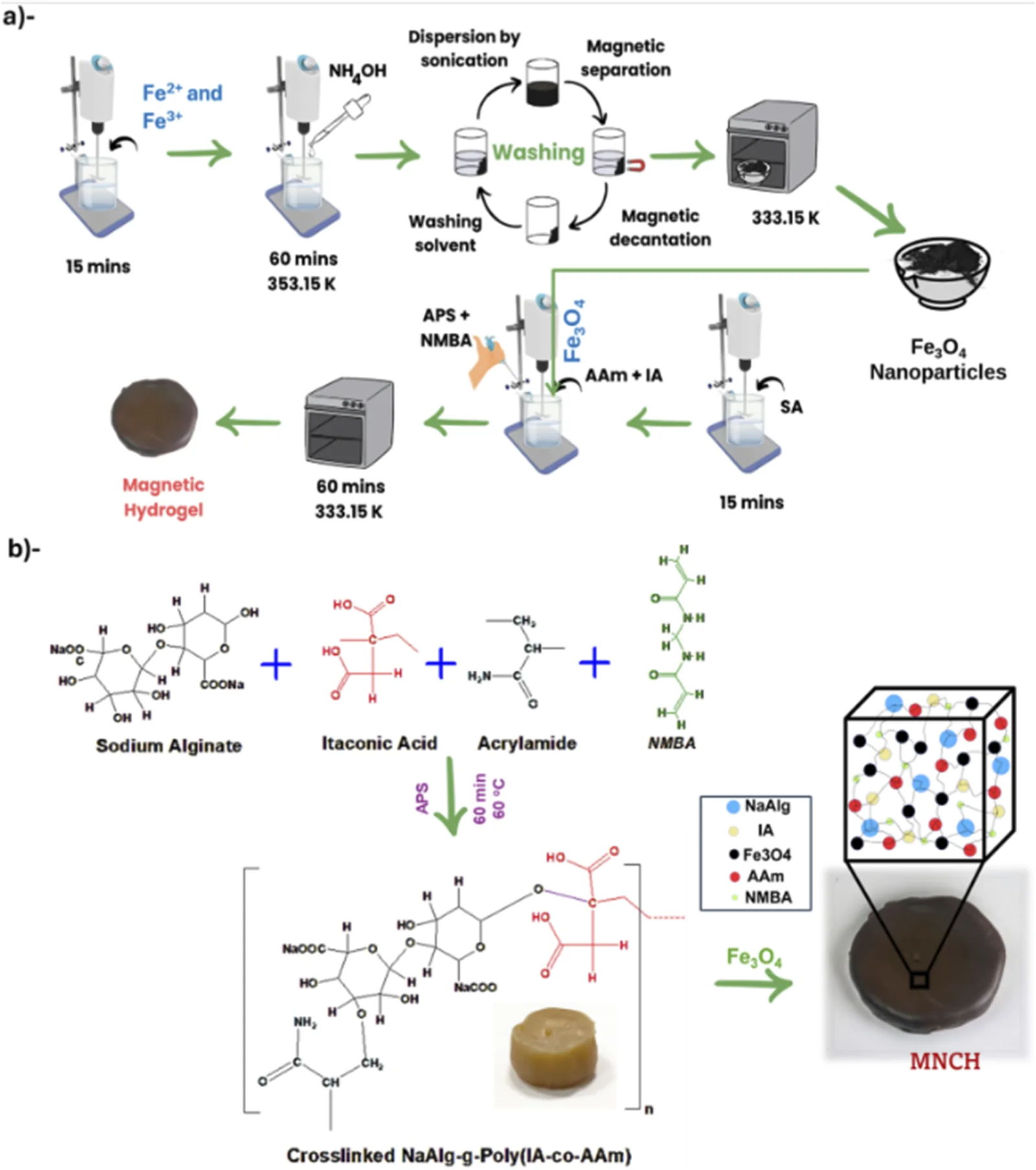
(a) Synthesis of Fe_3_O_4_ and Fe_3_O_4_ embedded magnetic nanocomposite hydrogel (MNCH). (b) Mechanism of synthesized MNCH. Reproduced from ref. 182 with permission from Elsevier, copyright 2025.

Hemdan *et al.*^183^ evaluates the performance of alginate-coated nano-iron oxide combined with copper-based metal–organic frameworks (Cu-BTC@Alg/Fe_3_O_4_) for removing rhodamine B dye from wastewater using both adsorption and photocatalysis. The composite's structure was confirmed through several analytical methods, including FTIR, XRD, SEM, and TEM. BET analysis showed the material has a large surface area of about 160 m^2^ g^−1^, which supports a high adsorption capacity of 200 mg g^−1^ and a removal efficiency of 97%. The composite also demonstrated strong photocatalytic activity, breaking down the dye into environmentally safe products, emphasizing its sustainable application for dye removal that shown in Fig. 11A.

**Fig. 11:**
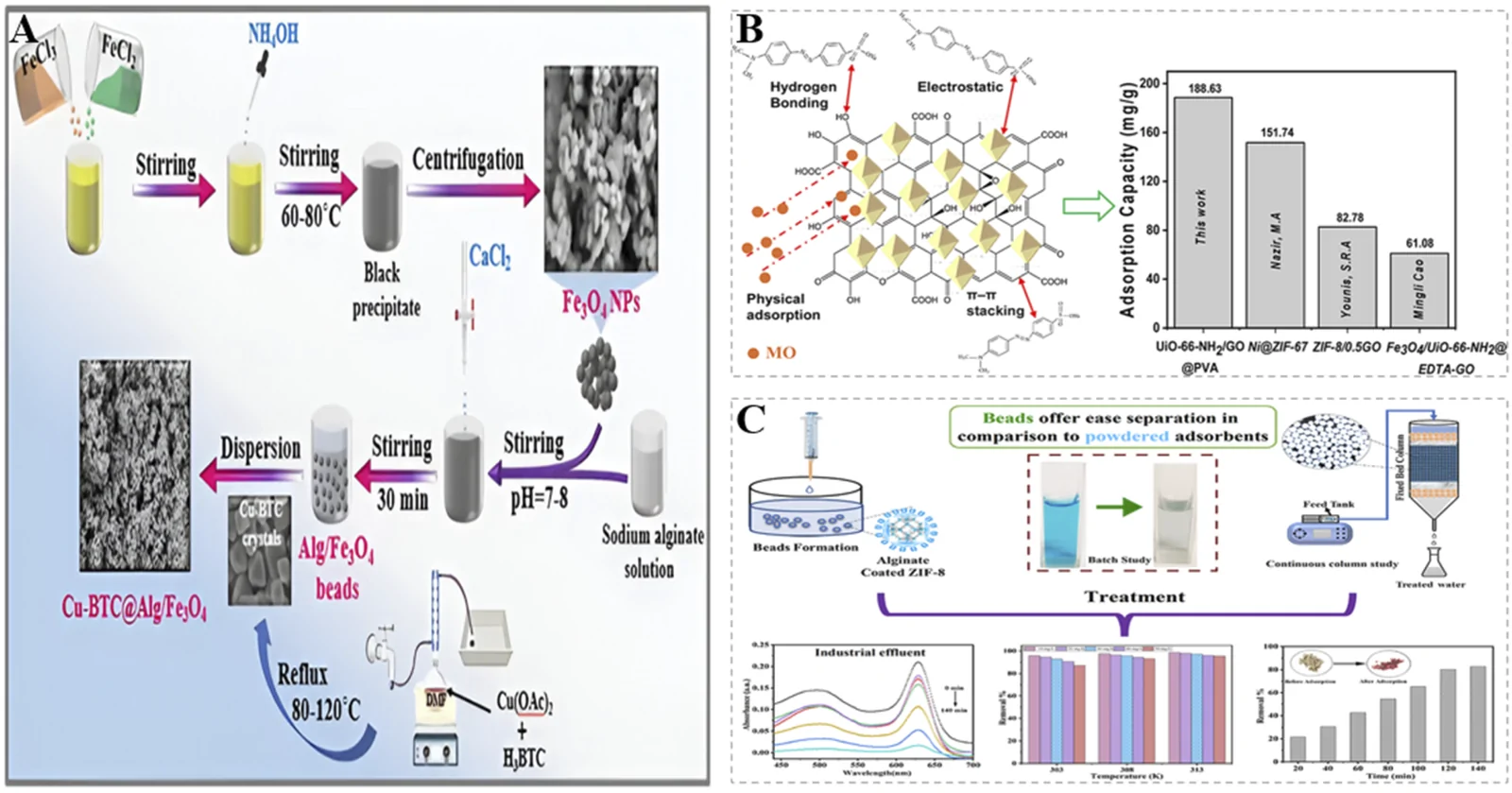
(A) Illustrates a schematic representation of the synthesis methods employed for the production of Cu-BTC@Alg/Fe_3_O_4_ composite. Reproduced from ref. 183 with permission from Elsevier, copyright 2023. (B) Illustrates a schematic representation of UiO66-NH_2_/GO/PVA. Reproduced from ref. 184 under the terms of the Creative Commons CC BY 4.0 licence, copyright 2025, the authors, published by American Chemical Society. (C) Schematic illustration of SA@ZIF-8 aiming to remove the MG and real industrial wastewater. Reproduced from ref. 185 with permission from Elsevier, copyright 2024.

The study of adsorption mechanisms revealed that both chemical and physical interactions are involved. Adsorption equilibrium was reached within 100 minutes for all starting concentrations, and the process followed a pseudo-second-order kinetic model (*R*^2^ ≈ 0.999). Among the tested isotherm models, Temkin and Dubinin–Radushkevich provided a better fit than the Langmuir model (*R*^2^ ≈ 0.98 and 0.96, respectively). Thermodynamic analysis indicated the process is spontaneous (negative Gibbs free energy of −6.40 kJ mol^−1^), endothermic (positive enthalpy of +24.3 kJ mol^−1^), and increases disorder at the interface (positive entropy of +82.06 kJ mol^−1^ K^−1^). Overall, the findings highlight the composite's effectiveness and potential for use in wastewater treatment applications.

V. Van Thang *et al.*^184^ reported a novel UiO-66-NH_2_/GO@PVA composite for the efficient removal of methyl orange (MO) from aqueous solutions, addressing the growing environmental concern associated with persistent synthetic dye contamination in water resources. The composite was fabricated by incorporating graphene oxide (GO) and amino-functionalized UiO-66-NH_2_ into a poly(vinyl alcohol) (PVA) matrix, resulting in a stable, highly porous adsorbent with favorable adsorption characteristics. The adsorption process exhibited a good fit to the Langmuir isotherm model (*R*^2^ = 0.9439), with a maximum adsorption capacity of 188.63 mg g^−1^. Under optimized conditions, a remarkable MO removal efficiency of 99.6% was achieved at an initial dye concentration of 100 mg L^−1^ and pH 5. Kinetic analysis showed excellent agreement with the pseudo-second-order model (*R*^2^ = 0.9930), suggesting that chemisorption plays a major role in the adsorption process. Thermodynamic analysis further indicated that MO adsorption was endothermic and spontaneous. Notably, the composite maintained more than 95% of its initial removal efficiency after four consecutive regeneration cycles, demonstrating good reusability and structural stability. Overall, the UiO-66-NH_2_/GO@PVA composite shows considerable potential as an efficient and environmentally friendly adsorbent for dye-contaminated wastewater treatment. Further investigations using real or simulated wastewater systems are warranted to assess its practical applicability under more complex environmental conditions Fig. 11B.

For mechanistic clarification, adsorption isotherm and kinetic models provide empirical descriptions of adsorption behavior and cannot alone confirm the molecular mechanism. Although Langmuir fitting is commonly associated with monolayer adsorption and pseudo-first-order/pseudo-second-order models with physical adsorption/chemisorption, respectively, these interpretations require complementary evidence. For Ca^2+^-crosslinked SA materials, dye uptake may involve electrostatic interactions, hydrogen bonding, surface interactions, and diffusion or partitioning within the hydrated polymer network. Therefore, the reported capacity of 4216.69 mg g^−1^, despite a BET surface area of 59.80 m^2^ g^−1^, can be interpreted cautiously as an apparent uptake rather than solely surface adsorption. The high capacity may partly arise from dye penetration and retention within the swollen SA matrix.

### 7.3 Sodium alginate@MOFs

Metal–organic frameworks (MOFs) are a versatile class of crystalline porous materials constructed from metal ions or metal clusters interconnected by organic ligands.^186–192^ Owing to their exceptionally high surface areas, tunable pore structures, abundant active sites, and tailorable chemical functionalities, MOFs have attracted considerable attention across a wide range of research fields.^193–195^ Their structural and chemical versatility has enabled their application in biochemical sensing,^196–199^ biological and biomedical activities,^200^ catalysis,^201–206^ energy storage and battery systems,^207^ gas storage and separation,^208–211^ and adsorption-based environmental remediation.^212–214^ In particular, the well-defined porous structures and modifiable metal centers and organic linkers of MOFs provide favorable platforms for molecular recognition, catalytic reactions, sensing, ion and gas storage, and the adsorption or removal of various contaminants.^215–222^ Consequently, MOFs have emerged as promising multifunctional materials for developing advanced technologies in environmental, energy, and biomedical applications.^223–225^ Despite these advantages, the practical application of pristine MOFs may be restricted by several limitations, including their fine-powder morphology, particle aggregation, limited mechanical stability, and difficulties in separation and recovery after use. Therefore, integrating MOFs with natural polymers has become an attractive strategy for improving their processability, structural stability, and recyclability while retaining their intrinsic porosity and adsorption properties. Among various biopolymers, SA is particularly attractive because it is a naturally derived, biodegradable, biocompatible, low-cost, and environmentally friendly polysaccharide containing abundant hydroxyl (–OH) and carboxylate (–COO^−^) groups. These functional groups can provide additional interaction sites for target pollutants and facilitate the immobilization or encapsulation of MOF particles within an alginate matrix.

The integration of MOFs with sodium alginate can therefore generate multifunctional SA@MOF composites, hydrogels, beads, membranes, and aerogels with improved structural integrity and easier handling compared with unsupported MOF powders. The porous framework of the MOF contributes a high density of accessible adsorption and catalytic sites, whereas the alginate matrix provides a flexible and hydrophilic support that can minimize particle aggregation and facilitate separation from treated water. Moreover, the combination of MOFs and alginate can generate synergistic interactions, including electrostatic attraction, hydrogen bonding, π–π interactions, pore filling, and surface complexation, depending on the structure of the MOF and the nature of the target pollutant. Such synergistic effects can enhance pollutant uptake and improve the practical applicability of MOF-based materials for wastewater treatment.

Garg *et al.*^185^ synthesized composite beads by incorporating zeolitic imidazolate framework-8 (ZIF-8) into a SA matrix using a simple dipping method. Comprehensive characterization, including XRD, FTIR, BET, TGA, and SEM-EDX analyses, was carried out to evaluate their structure and properties. SEM images verified that SA uniformly coated the cubic ZIF-8 particles, supporting the formation of an efficient adsorbent. The performance of the SA@ZIF-8 beads was assessed against synthetic malachite green (MG) dye and real industrial wastewater using both batch and fixed-bed column setups. The beads achieved a maximum removal efficiency of 95.5% at pH 6 within 120 minutes. FTIR and SEM results further confirmed successful adsorption of MG onto the bead surfaces. Adsorption behavior was best described by the Freundlich isotherm model (*R*^2^ > 0.99), outperforming the Langmuir model (*R*^2^ > 0.96), while kinetic studies indicated that the process followed a pseudo-second-order model. Intraparticle diffusion analysis also contributed to understanding the underlying adsorption mechanism. The study additionally demonstrated the practical effectiveness of the beads in treating real dye-industry effluent. Compared with powdered ZIF-8, the SA-coated ZIF-8 beads showed superior selectivity, stability, reusability, and operational convenience, highlighting their strong potential for industrial-scale dye removal and broader wastewater treatment applications (Fig. 11C).

Ye *et al.*^226^ developed a novel MOF-based adsorbent, calcium polyvinyl alcohol@UiO-66, using environmentally friendly polymers—SA and polyvinyl alcohol—to form double-network gel spheres through cross-linking. UiO-66 was subsequently grown on the surface of these gel spheres, yielding the final CA/PVA@UiO-66 composite. The resulting material features a high surface area (17.4 m^2^ g^−1^) and a mesoporous–microporous hierarchical structure. It demonstrated excellent performance in removing methylene blue (MB) from aqueous solutions, achieving an actual maximum adsorption capacity of 275.8 mg g^−1^—exceeding that of many previously reported adsorbents. Impressively, the material maintained 93.9% of its original adsorption capacity after 10 regeneration cycles. Adsorption behavior followed the Redlich–Peterson isotherm and pseudo-first-order (PFO) kinetic model. Results from N_2_-sorption analysis, MB adsorption experiments, and model fitting indicated that the process involves complex heterogeneous diffusion with both chemisorption and physisorption occurring simultaneously. Thermodynamic evaluation confirmed that the adsorption is endothermic and spontaneous at 298 K, with higher temperatures further enhancing adsorption capacity. Overall, the gel-based CA/PVA@UiO-66 adsorbent exhibits strong dye-removal efficiency and excellent reusability, making it a promising candidate for wastewater purification applications (Fig. 12A).

**Fig. 12:**
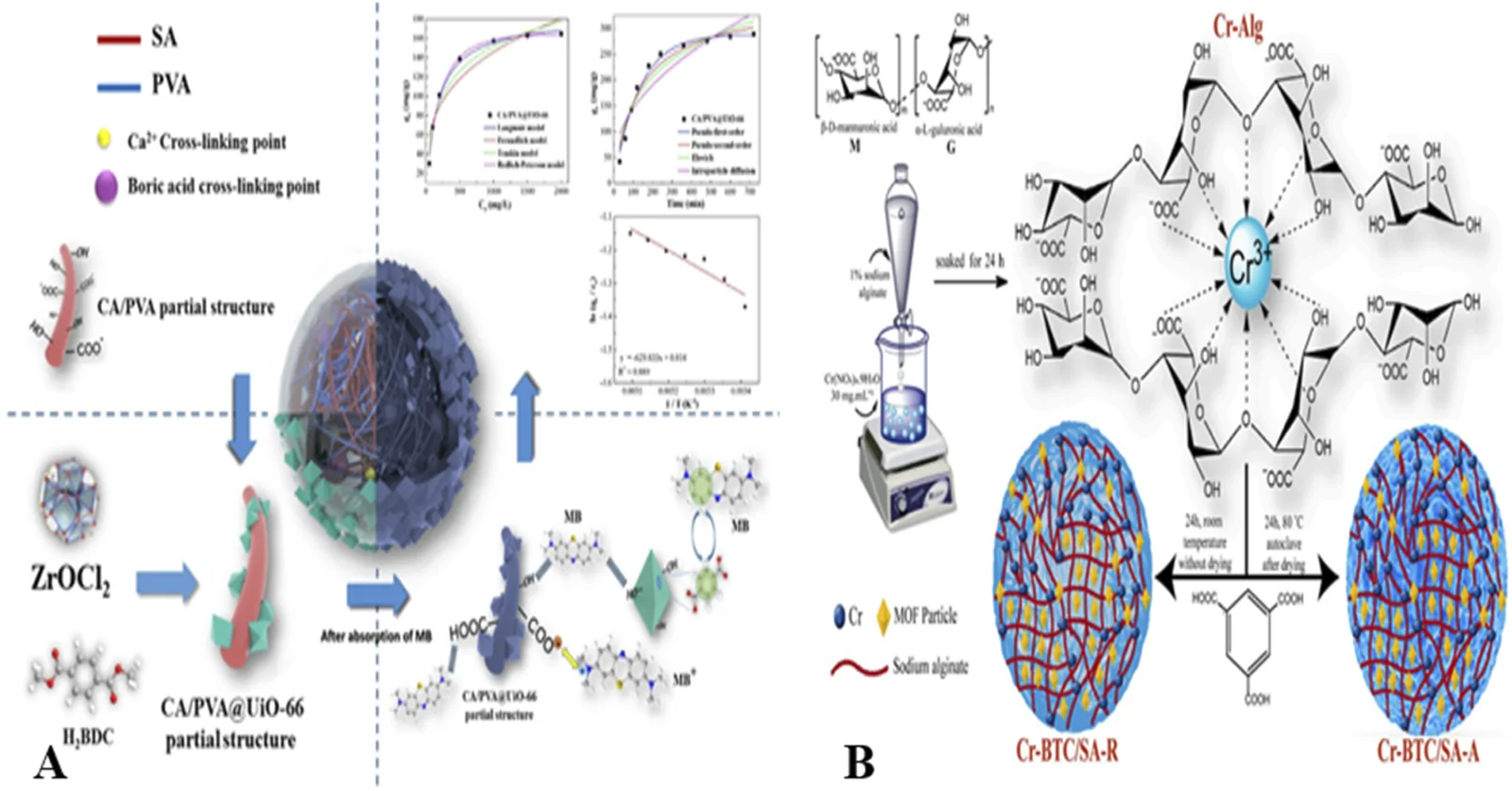
(A) Schematic illustration of CA/PVA@UiO-66 composite to remove the MB. Reproduced from ref. 226 with permission from Elsevier, copyright 2024. (B) Schematic illustration of the Cr-Alg, Cr-BTC/SA-R, and Cr-BTC/SA-A preparation procedure. Reproduced from ref. 227 with permission from Elsevier, copyright 2024.

Sonmez *et al.*^227^ developed two types of macroscopic Cr-MOF-alginate composite beads using an *in situ* growth technique for the removal of methylene blue (MB) from aqueous solutions. Characterization analyses confirmed the successful formation of two distinct Cr-MOF-Alg composites, each exhibiting different MB adsorption behaviors depending on the synthesis route. Optimal operating conditions were determined as follows: 0.3 g adsorbent dose, pH 10, 120 minutes contact time, an initial MB concentration of 10 mg L^−1^, and a temperature of 25 °C. Under these conditions, maximum MB removal efficiencies reached 90.33% for Cr-Alg, 93.42% for Cr-BTC/SA-R, and 94.06% for Cr-BTC/SA-A.

Isotherm and kinetic modeling indicated that adsorption onto both Cr-Alg and Cr-MOF-Alg beads involves a combination of chemisorption and physisorption processes. Thermodynamic results further demonstrated that the adsorption is both endothermic and spontaneous. Overall, these findings highlight the potential of Cr-MOF-alginate composites as effective adsorbents for dye-contaminated wastewater (Fig. 12B).

## 8. Industrial-scale implementation and challenges

Large scale applications of SA-based solutions may present issues like quality and performance of the composites since they form part of an industry related chain. Such factors that directly influences the efficiency of the material includes: the dispersion of nanoparticles both throughout the alginate matrix, the enhancement of the adsorption capacity of alginate, and the implementation of proper regeneration procedures for the reusability of the adsorbent. In a pilot scale study in India, with SA-based composites treatment capacities up to 50 000 liters of dye polluted water per day were feasible; however, general dye wastewater conditions significantly impacted treated water quality. The scalability of these technologies is based on realizing new material processing methods and manufacturing costs.

## 9. Practical considerations for real textile wastewater

Although model-dye solutions are useful for evaluating adsorption mechanisms and removal capacity, real textile wastewater contains salts, multivalent ions, surfactants, chelating agents, dissolved organic matter, and other competing species that may substantially affect adsorption and material stability. High Na^+^ concentrations can alter ionic interactions within the Ca^2+^-alginate network and influence electrostatic adsorption, while Mg^2+^, Fe^3+^, and Al^3+^ may compete for carboxylate sites and modify the crosslinked structure. Chelating agents such as EDTA and polyphosphates may complex Ca^2+^ and promote partial decrosslinking, swelling, and loss of mechanical integrity, whereas surfactants and dissolved organic constituents may compete with dyes for accessible adsorption sites.

Therefore, dye removal should be distinguished from overall organic removal, and parameters such as COD/TOC should be considered when evaluating treatment performance. For practical application, batch adsorption capacity should also be supported by continuous-flow studies addressing breakthrough behavior, mass transfer, hydraulic resistance, bead swelling, compressibility, and pressure drop. Furthermore, regeneration conditions must be carefully controlled because repeated treatment cycles may cause Ca^2+^ leaching, structural weakening, and loss of adsorption capacity. Accordingly, long-term applicability should be assessed through repeated adsorption–regeneration cycles using increasingly realistic wastewater matrices, with monitoring of dye removal, Ca^2+^ release, mass loss, swelling, mechanical stability, and hydraulic performance. Overall, these considerations highlight that adsorption results obtained with model dyes represent intrinsic material performance, whereas validation with multicomponent and authentic textile wastewater is necessary to establish the practical durability and industrial applicability of Ca^2+^-crosslinked SA materials.

## 10. Future perspectives and discussion

This review highlights the significance of SA-based materials for textile water treatment applications. Currently, many challenges remain to be thoroughly investigated for advancements in this field. Therefore, there are some recommendations for future researchers.

Research focused at optimizing existing SA-based composites and innovating new ones with unique characteristics tailored for specific applications represent another area to be focused. There are free hydroxyl and carboxyl groups present along the polymer chain backbone in SA. Future investigations into chemical modifications targeting these two functional groups can potentially produce SA-based materials with enhanced properties. By blending SA with functional polymers and making composite with inorganic nano-materials, the adsorption capacities of materials can be enhanced. Researchers need to address properties such as large-scale availability, stability, regeneration capacity, mechanical strength for further application. Furthermore, current studies primarily focus on materials having porous structures and internal functional groups because of this trend there is lack of understanding of complex mechanisms. There is more than one type of pollutants present when dealing with wastewater applications such as viruses, bacteria, organic compounds, microplastics, pharmaceuticals, heavy metals. So, for dealing such type of wastewater treatment applications researchers should tailor the SA-based material in a way to remove multiple type of pollutants from water.

## 11. Conclusion

This review examines the adsorptive performance and physicochemical properties of various sodium alginate (SA)-based materials. It provides a comprehensive overview of the use of SA-based nanocomposites as effective materials for textile wastewater treatment, highlighting key aspects such as the molecular structure and physical properties of alginate, methods for SA modification, and the development of SA nanocomposites incorporating polymers, clays, metals, and metal oxides. In addition, the review discusses the fabrication of electrospun sodium alginate-based nanofibers and crosslinked SA nanocomposites. SA is an environmentally friendly, biodegradable, bioavailable, and cost-effective material, making it well suited for a wide range of wastewater treatment applications. Modification of SA significantly improves its mechanical strength and physicochemical performance compared with the unmodified polymer. A thorough understanding of alginate structure and modification strategies is therefore essential for the rational design, development, and effective application of modified SA-based materials in wastewater treatment systems.

## Conflicts of interest

There are no conflicts to declare.

## Data Availability

No new data were generated or analyzed in this study. All information discussed in this review was obtained from publicly available published literature, which is appropriately cited in the manuscript.
